# Comparative clinical outcomes of dronedarone and sotalol in Asian patients with atrial fibrillation: a nationwide cohort study

**DOI:** 10.1038/s41598-020-73115-y

**Published:** 2020-09-30

**Authors:** So-Ryoung Lee, Eue-Keun Choi, Ji-Hyun Kim, Jung-Ae Kim, Tae-Yeon Kwon, Young Eun Lee, Seil Oh

**Affiliations:** 1 Division of Cardiology, Department of Internal Medicine, Seoul National University Hospital, 101 Daehak-ro, Jongno-gu, Seoul, 03080 Republic of Korea; 2 Medical Affairs, Sanofi-Aventis Korea, Seoul, Republic of Korea; 3 Real World Insights, IQVIA Korea, Seoul, Republic of Korea

**Keywords:** Cardiology, Outcomes research

## Abstract

We aimed to evaluate the effectiveness and safety of dronedarone versus sotalol in real-world practice in Asian patients with atrial fibrillation (AF). Using the Korean nationwide claims database from August 2013 to December 2016, we identified patients with AF recently prescribed dronedarone or sotalol and analyzed the hospitalization risk and all-cause death until December 2017. Overall, 3119 and 1575 patients treated with dronedarone and sotalol, respectively, were included. After propensity score weighting, no significant differences were observed between the treatment groups. Dronedarone use was associated with a lower risk of all-cause hospitalization than sotalol use (hazard ratio [HR], 0.79; 95% confidence interval [CI], 0.70–0.88). The dronedarone group demonstrated a significantly lower risk of cardiovascular (CV) hospitalization than the sotalol group (HR 0.62, 95% CI 0.53–0.72); however, no significant difference was observed in non-CV hospitalization. No difference in the risk of all-cause death was observed between groups. The dronedarone group was significantly less likely to receive nonpharmacological treatment for AF than the sotalol group (HR 0.63, 95% CI 0.51–0.77). In a large-scale population of Asian patients with AF, dronedarone was associated with a lower risk of CV hospitalization and a lower need for nonpharmacological treatment for AF than sotalol.

## Introduction

Atrial fibrillation (AF) is the most common cardiac arrhythmia encountered in clinical practice, with a growing prevalence globally, including Asia^1,2^. With the increasing number of patients presenting AF, the care burden, including hospitalization and medical costs, has increased^3^. Patients with AF present higher risks of stroke, heart failure, and mortality than the general population^4^. Adequate oral anticoagulation is the cornerstone of AF management^5^, which reduces the risk of stroke in high-risk patients with AF. Furthermore, regulating AF itself reportedly improves the quality of life and reduces hospitalization owing to heart failure^6,7^. According to a recent study, the number of hospitalizations for AF has been continuously increasing over the last 10 years^3^.

Pharmacological rhythm control is the first-line treatment for AF^5^. Dronedarone is an antiarrhythmic drug (AAD) used for the treatment of AF, significantly reducing cardiovascular (CV) events or death when compared with placebo, mainly owing to reduced hospitalizations for AF and arrhythmia-related mortality^8^. Reportedly, dronedarone has fewer adverse effects related to organ (thyroid, lung, and liver) toxicities than amiodarone^9^. Notably, guidelines have recommended the use of dronedarone for rhythm control therapy in symptomatic AF patients without heart failure^5^. Sotalol is an AAD demonstrating a similar recommendation guideline to dronedarone. Although there are no direct comparisons between dronedarone and sotalol to date, recent mixed treatment comparisons from clinical trial data have provided insights regarding the comparative effectiveness and safety of these two AADs, revealing that dronedarone therapy was associated with lower risks of proarrhythmic events and all-cause death than sotalol treatment^10^. A real-world observational study has reported findings consistent with those observed in clinical trials^11^. However, data regarding the effectiveness and safety of dronedarone when compared with sotalol remains limited, especially in Asian patients with AF in the real-world setting.

In this study, we aimed to compare the clinical outcomes of dronedarone and sotalol among patients with AF in real-world clinical practice by using a Korean nationwide observational cohort.

## Methods

### Data sources

This was a nationwide retrospective observational cohort study utilizing Korean National Health Insurance claims data from the Health Insurance Review and Assessment (HIRA) database, which contains demographic and medical claims information of more than 50 million Koreans^12,13^. South Korea has a single-payer, universal, and compulsory health insurance system, covering approximately 98% of the entire Korean population. As the database contains patients’ diagnoses, procedures, treatments, surgeries, and prescription medications, it represents the entire South Korean population, with advantages for generalization across the entire population. Diagnoses were coded using the International Classification of Diseases, Tenth Revision (ICD-10), Clinical Modification codes. The claims database based on the Korean HIRA did not include the laboratory data^12^. This study was approved by the Institutional Review Board of Seoul National University Hospital (E-1811-002-982). The need for informed consent was waived by the review board as the data of each patient in the HIRA database were de-identified and encrypted to protect patient privacy. All methods were performed in accordance with the relevant guidelines and regulations.

### Study design

The detailed inclusion/exclusion criteria and the patient enrollment process are presented in Supplementary Methods, Supplementary Table S1, and Supplementary Fig. S1. Patients who received a target AAD, dronedarone or sotalol, with 7 or more days of drug supply during the enrollment period (August 1, 2013, to December 31, 2016) were included. Among them, patients who had more than one claim with a diagnosis code of AF during 36 months before the first dronedarone or sotalol prescription were included. Patients aged < 18 years were excluded. To include only “new users” of dronedarone or sotalol, we excluded patients who were prescribed target AADs during the screening period (12 months before the first prescription of target AADs). During the screening period, patients with prevalent cancer, end-stage renal disease, and any diagnoses related to contraindications in the Korean label recommendations of dronedarone or sotalol were identified and excluded from the analysis^14^. Additionally, patients prescribed a medication contraindicated with dronedarone or sotalol during the screening period were excluded^14^. Relevant diagnosis codes, drug codes, and detailed operational definitions for the exclusion criteria are presented in Supplementary Table S1. Finally, 4694 patients with AF recently prescribed dronedarone or sotalol were identified during the study period (Supplementary Fig. S1).

### Covariates

The potential confounders and demographic characteristics during the baseline period were considered as covariates. Patient demographic characteristics, comorbidities, and concomitant medications were ascertained from the Korean HIRA database. All covariates were assessed during the screening period (12 months before the index date). Supplementary Table S2 summarizes the definitions of codes of comorbidities. The comorbidities included hypertension, diabetes, dyslipidemia, congestive heart failure, ischemic heart disease, myocardial infarction (MI), peripheral artery disease, stroke, thromboembolism, transient ischemic attack (TIA), and chronic lung disease^2^. In Supplementary Methods, we provided additional information and discussion regarding the reliability and validation of operational definitions. Previous papers have reported the reliable accuracy of ICD-10 diagnostic codes in Korean National Health claims data^15–20^. The CHA_2_DS_2_-VASc score for assessing stroke risk and the modified Charlson Comorbidity Index (mCCI) for estimating the burden of comorbidities were calculated, combining covariate information^4,21^. The detailed definitions of the number of baseline AADs, type of baseline AADs, hospital type, insurance type, and index year as covariates are presented in Supplementary Methods.

### Study outcomes and follow-up

The index date was defined as the first prescription date of the target AAD. To compare the clinical outcomes of dronedarone versus sotalol, the primary, secondary, and exploratory secondary outcomes were evaluated within the study period. The detailed definitions of clinical outcomes are presented in Supplementary Table S3. The primary outcome was defined as a composite of CV hospitalization and all-cause death^8^. The secondary outcomes were defined as a CV or non-CV hospitalization, all-cause hospitalization, CV or non-CV death, all-cause death, stroke, and MI. To examine treatment compliance, we evaluated drug persistence and adherence as secondary outcomes. The definitions of persistence and adherence are presented in Supplementary Methods. For exploratory secondary outcomes, nonpharmacological rhythm control was defined as the composite of electrical cardioversion and AF catheter ablation during the study period. Hospitalization owing to potential safety signals, including acute liver injury (ALI), thyroid disorder, and interstitial lung disease (ILD), was further investigated to clarify the potential risks as suggested in previous reports^11,14^. The follow-up period ended when patients discontinued the index drug for > 30 days, the study outcome was achieved, or the study period ended (December 2017), whichever came first.

### Statistical analysis

All key parameters were descriptively analyzed and are presented as mean, standard deviation, minimum, maximum, and median values when continuous, and as number and percentage when categorical. Descriptive statistics of the study groups were compared using the t-test for continuous variables and the Chi-square test for categorical variables.

The inverse probability of treatment weight (IPTW) was estimated from propensity scores, as a possibility existed that the initial AAD would be chosen based on patient demographics and baseline characteristics. The propensity score was defined as a patient’s treatment selection probability, conditional on observed baseline characteristics. For propensity score estimation, patient demographics and baseline characteristics expected to affect the AAD selection were considered in logistic regression. The IPTW was constructed using the inverse of the estimated propensity score. However, the estimated IPTW tended to have extreme outliers, and the large weight, including the extreme outliers, presented disadvantages such as creating an unstable pseudo-population that was heavily dependent on a single or few individuals and increasing the variance. Moreover, an inflated pseudo study population with a considerably large weight tended to reject the null hypothesis frequently owing to the doubled sample size. Conversely, a stabilized weight tended to produce estimates with smaller variance, resulting in pseudo-data with a similar sample size to the observed data. Therefore, in this study, the stabilized IPTW was considered to obtain robust results. The stabilized IPTW was estimated by multiplying the IPTW by the baseline probability of treatment selection without covariates. For example, the probability function of the stabilized IPTW for a patient treated with dronedarone was equal to p(z = dronedarone)/p(z = dronedarone|x = covariates)^22^. The IPTW was estimated based on age, sex, CHA_2_DS_2_-VASc score, comorbidities, concomitant medications, number of baseline AADs, baseline AADs, hospital type, insurance type, and index year, as it could affect the AAD treatment selection, and was stabilized by multiplying the estimate with the baseline probability of treatment selection. Additionally, the comparability of the pseudo-population with the IPTW was evaluated using absolute standardized mean differences (ASDs). Differences in ASDs of < 10% were considered to indicate well-balanced characteristics^23^.

For clinical outcomes, crude and weighted incidence rates were calculated using crude and weighted event numbers during the follow-up period, divided by 100 person-years at risk in each clinical outcome. Using survival analysis, the risk of each clinical outcome with dronedarone use versus sotalol use was compared by applying the Kaplan–Meier method (log-rank test) with IPTW and weighted Cox proportional hazard regression models with IPTW. The sotalol group was used as a reference group in the Cox regression analysis. The methods for examining drug persistence and adherence are described in Supplementary Methods.

All statistical tests were two-sided with a significance level of 0.05. The statistical analysis for this paper was generated using SAS software (version 9.3). Copyright 2016 SAS Institute Inc. SAS and all other SAS Institute Inc. product or service names are registered trademarks or trademarks of SAS Institute Inc., Cary, NC, USA.

### Sensitivity analyses

Considering the differences in the follow-up duration between the two groups, we performed sensitivity analyses restricting the follow-up period to 6 months and 12 months. In these analyses, patients were censored at 6 months or 12 months after the index date.

### Subgroup analyses

The comparisons between dronedarone and sotalol were supplemented by subgroup analyses according to the following subgroups: age strata, sex, CHA_2_DS_2_-VASc score, number of baseline AADs, mCCI, and the presence of heart failure. For subgroup analyses, we used multivariable Cox proportional hazard regression models using all variables included for propensity score calculation. The statistical significance of the interaction between treatments was defined as a p-interaction value of < 0.1.

## Results

### Baseline characteristics

A total of 4694 patients were eligible for this study (3119 patients treated with dronedarone and 1575 patients treated with sotalol). Before IPTW estimation, patients in the dronedarone group were older and presented higher CHA_2_DS_2_-VASc scores and mCCI than those in the sotalol group (Table 1). After IPTW estimation, there were no significant differences in baseline characteristics between the two treatment groups (ASDs for all covariates < 0.1; Table 1 and Supplementary Fig. S2). After IPTW, the mean age of the dronedarone and sotalol groups were 62.7 ± 11.9 and 61.9 ± 12.9 years, respectively (ASD = 0.04); the mean CHA_2_DS_2_-VASc score of the dronedarone and sotalol groups were 2.66 ± 1.66 and 2.70 ± 1.67, respectively (ASD = 0.02) (Table 1). The mean follow-up duration was 335 days (median 177 days, interquartile range [IQR] 56–501 days): 368 days (median 201 days, IQR 59–549 days) in the dronedarone group and 270 days (median 140 days, IQR 45–412 days) in the sotalol group (p < 0.001).

**Table 1:** Baseline characteristics of the study population, before and after IPTW.

	Before IPTW			After IPTW		
	Dronedarone (n = 3119)	Sotalol (n = 1575)	ASD	Dronedarone (N = 3123)	Sotalol (N = 1571)	ASD
**Age, years**	63.2 ± 11.9	60.7 ± 12.9	0.20	62.7 ± 11.9	61.9 ± 12.9	0.04
Median (IQR)	63 (55–72)	62 (53–70)		63 (55–71)	63 (54–71)	
< 65	53.8	58.1		54.9	54.1	
65–74	27.7	28.9		28.4	29.2	
≥ 75	18.5	13.0		16.7	16.7	
**Men**	65.3	66.0	− 0.01	65.6	65.5	0.00
**CHA**_**2**_**DS**_**2**_**-VASc score**	2.72 ± 1.66	2.50 ± 1.62	0.13	2.66 ± 1.66	2.70 ± 1.67	0.02
Median (IQR)	3 (1–4)	2 (1–4)		2 (1–4)	2 (1–4)	
**Charlson Comorbidity Index**	1.32 ± 1.49	1.21 ± 1.43	0.08	1.29 ± 1.47	1.32 ± 1.51	0.01
Median (IQR)	1 (0–2)	1 (0–2)		1 (0–2)	1 (0–2)	
**Number of baseline AADs**	0.68 ± 0.70	0.70 ± 0.73	0.02	0.69 ± 0.71	0.70 ± 0.70	0.01
Median (IQR)	1 (0–1)	1 (0–1)		1 (0–1)	1 (0–1)	
0	43.4	44.4		43.2	42.2	
1	46.3	43.4		45.8	46.8	
≥ 2	10.2	12.5		11.0	10.9	
**Baseline AADs**						
Flecainide	15.8	23.9	0.20	19.1	19.5	0.01
Propafenone	17.6	16.3	0.03	17.1	17.0	0.00
Pilsicainide	8.0	4.7	0.14	6.9	6.7	0.01
Amiodarone	26.9	25.0	0.05	26.3	27.0	0.01
**Concomitant medication**						
ß-blocker	50.1	49.2	0.02	50.2	51.2	0.02
CCBs	29.2	33.3	0.09	30.6	31.1	0.01
Digoxin	4.8	8.6	0.15	5.8	5.9	0.00
ACE inhibitors or ARB	7.9	7.9	0.00	7.8	7.9	0.00
Statins	53.2	44.6	0.17	50.4	50.6	0.00
Warfarin	29.1	38.0	0.19	32.2	33.0	0.02
Factor Xa inhibitors	13.4	10.7	0.08	12.5	12.5	0.00
Aspirin	46.8	42.9	0.08	45.7	46.5	0.02
**Hospital type**						
Tertiary hospital	58.0	76.1	0.39	64.2	64.1	0.00
General hospital	37.6	21.1	0.37	31.9	32.0	0.00
Hospital or general practitioner	4.3	3.1	0.07	3.9	3.9	0.00
**Insurance type**						
Health insurance	96.4	96.2	0.01	96.4	96.4	0.00
Medical aid	3.6	3.8		3.6	3.6	
**Index year**						
2013	12.3	10.5	0.06	11.7	11.9	0.01
2014	27.5	27.2	0.01	27.3	27.0	0.01
2015	27.6	34.6	0.15	30.0	30.6	0.01
2016	32.6	27.6	0.11	30.9	30.5	0.01

*ASD* absolute standardized differences, *IQR* interquartile range, *IPTW* inverse probability of treatment weighting, *AADs* antiarrhythmic drugs, *CCBs* calcium channel blockers, *ACE* angiotensin-converting enzyme, *ARBs* angiotensin II receptor blockers.

During follow-up, the crude incidence rates of CV, non-CV, and all-cause hospitalization were 19.95 (n = 756), 24.74 (n = 861), 43.48 (n = 1,396) per 100 person-years, respectively; in total study population, the crude incidence rates of CV, non-CV, and all-cause death were 0.12 (n = 5), 0.57 (n = 24), and 0.69 (n = 29), respectively.

### Primary outcomes

**Figure 1:**
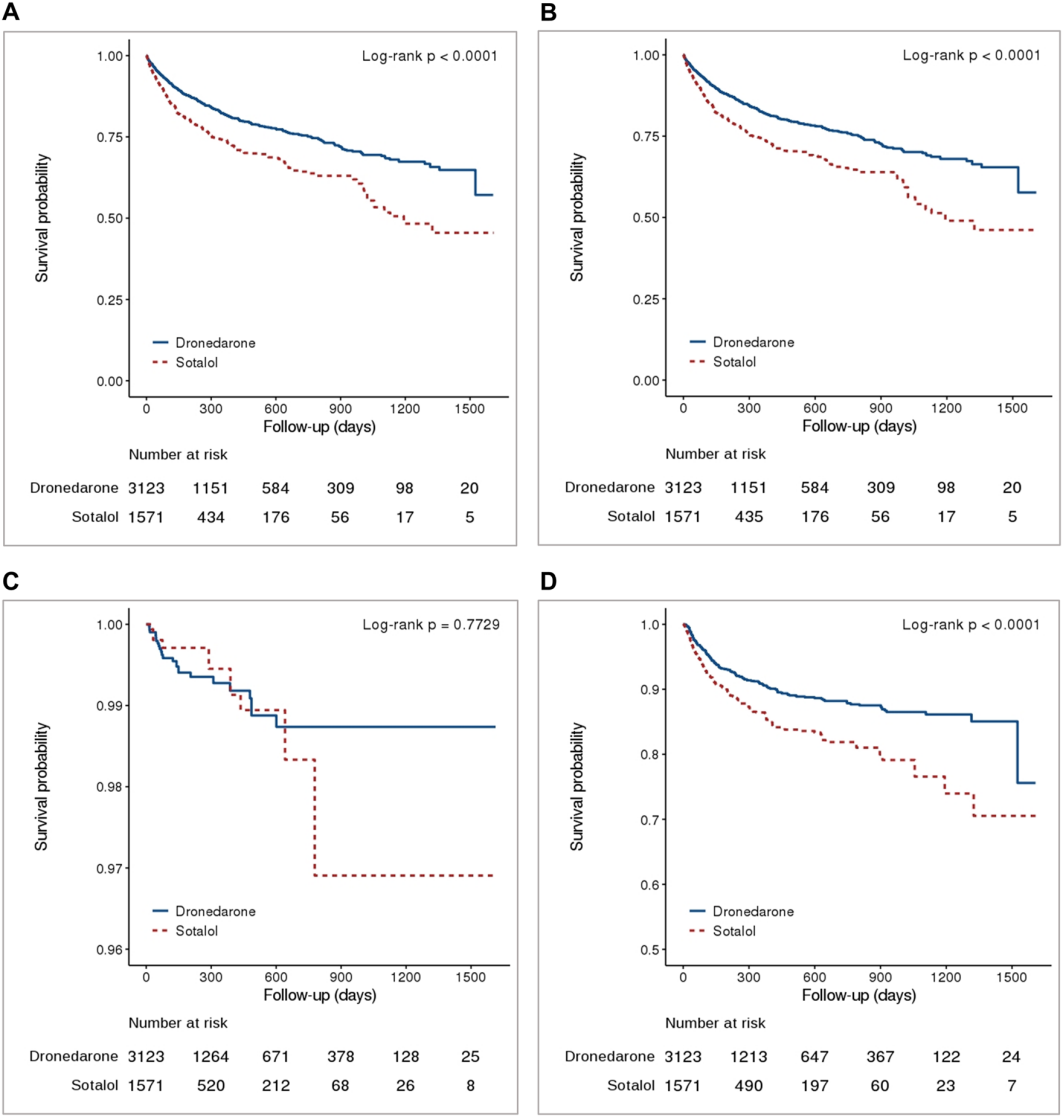
Weighted cumulative Kaplan–Meier curves of dronedarone versus sotalol groups in clinical outcomes (**A**) Primary outcome [Composite of CV hospitalization and all-cause death]; (**B**) Secondary outcome [CV hospitalization]; (**C**) Secondary outcome [All-cause death]; and (**D**) Exploratory outcome [Non-pharmacologic rhythm control]. CV, indicates cardiovascular; IPTW was estimated using logistic regression with patient age, sex, CHA_2_DS_2_-VASc score, mCCI, number of baseline AADs, type of baseline AADs, comorbidity, concomitant drugs, hospital type at index date, insurance type and index year.

The number of events, crude incidence rates, and weighted incidence rates of primary and secondary outcomes in the dronedarone and sotalol groups are presented in Supplementary Table S4 and weighted Kaplan–Meier curves are presented in Fig. 1 and Supplementary Fig. S3.

#### Primary outcome: composite of CV hospitalization and all-cause death

**Figure 2:**
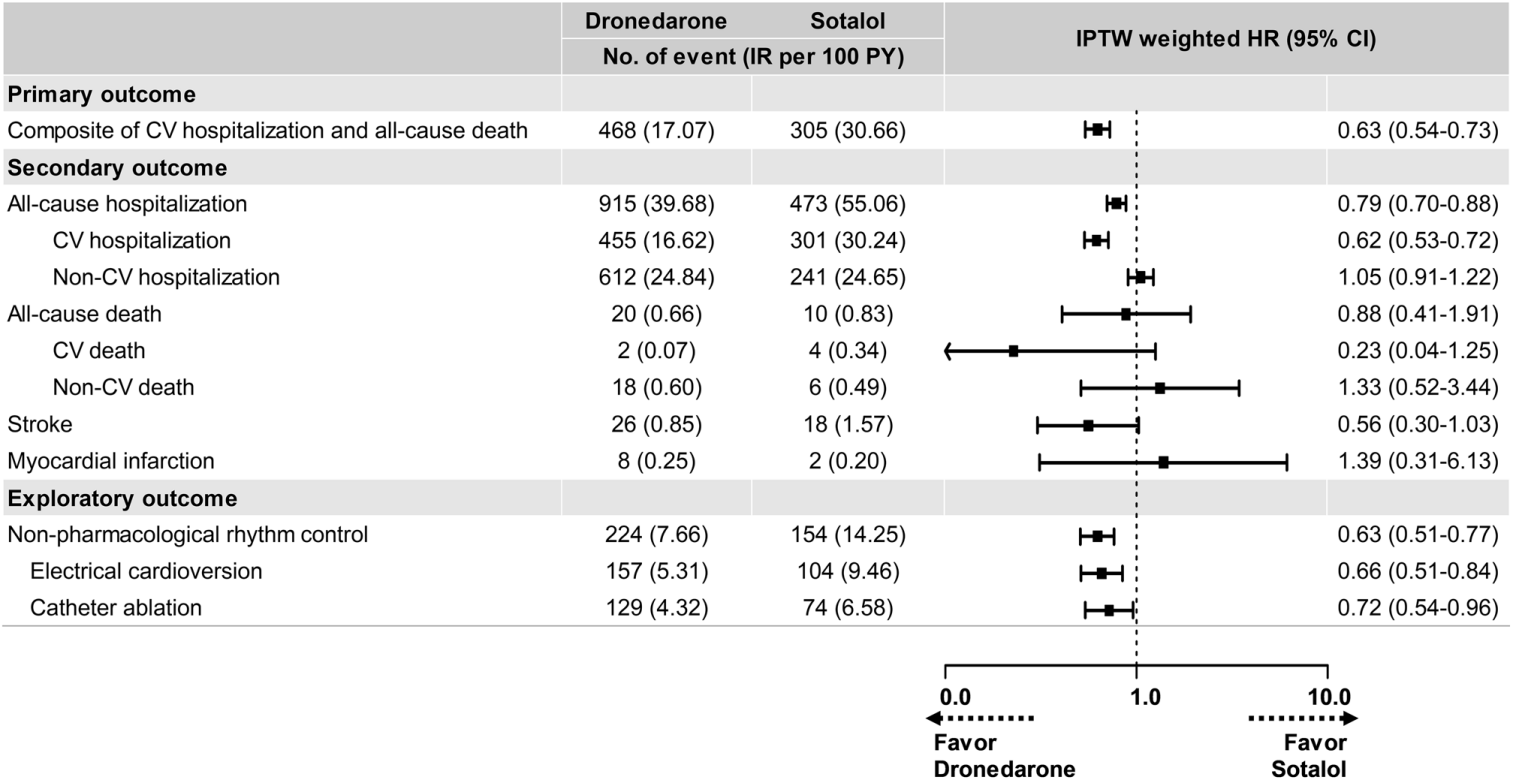
Dronedarone versus sotalol: Hazard ratios of clinical outcomes**.** The sotalol group was used as a reference group. *IPTW* indicates inverse probability of treatment weight, *IR* incidence rate, *PY* person-year, *HR* hazard ratio, *CI* confidence interval, *CV* cardiovascular. IPTW was estimated using logistic regression with patient age, sex, CHA_2_DS_2_-VASc score, mCCI, number of AADs in baseline, type of AADs in baseline, comorbidity, concomitant drugs, hospital type at index date, insurance type and index year.

Dronedarone use was associated with lower risks of the composite of CV hospitalization and all-cause death (hazard ratio [HR] 0.63, 95% confidence interval [CI] 0.54–0.73) (Fig. 2). The lower risk of primary outcome in dronedarone group was mainly driven by the lower risk of CV hospitalization of the dronedarone group compared to sotalol group.

#### Subgroup analyses for the primary outcome

**Figure 3:**
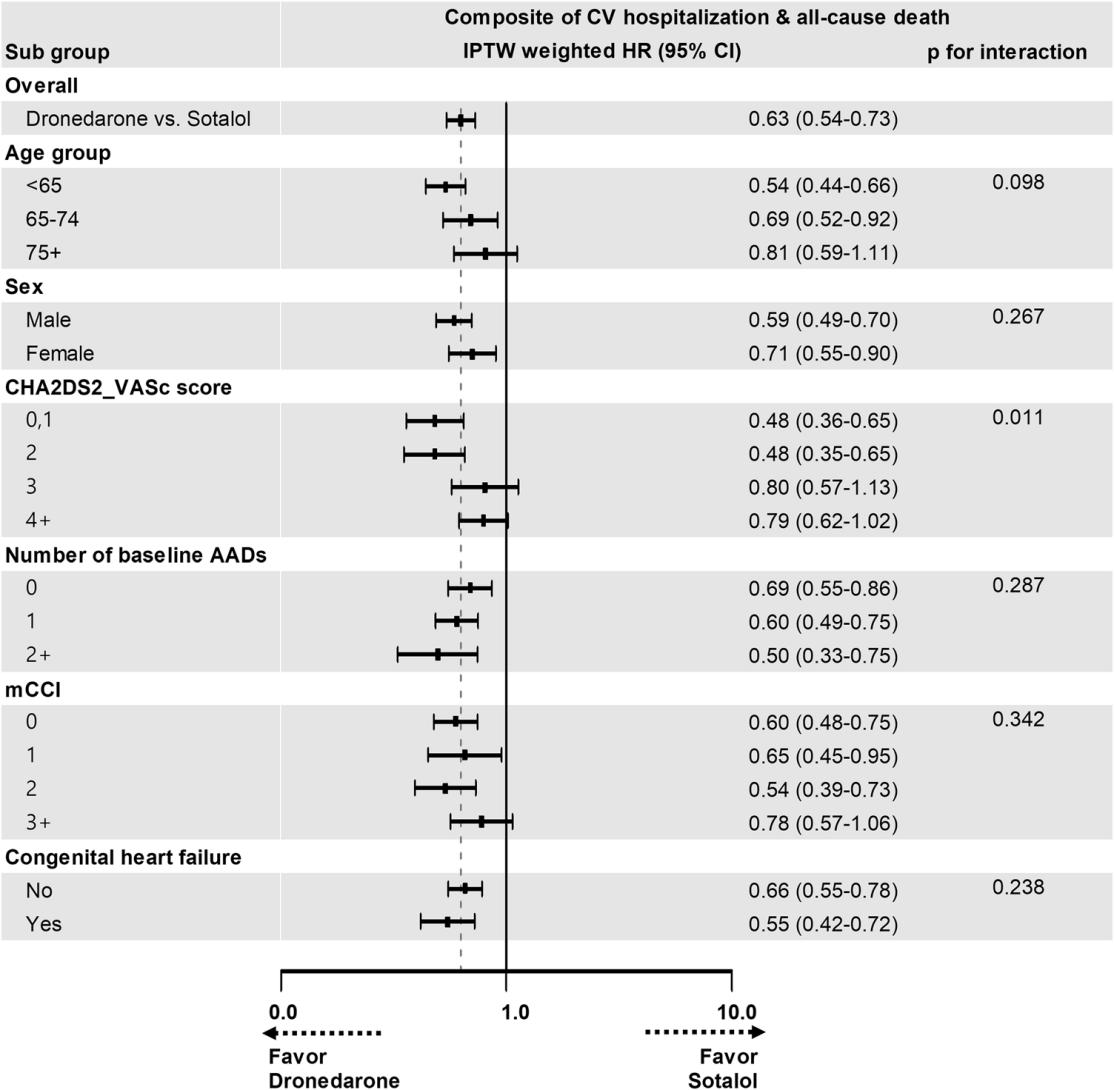
Hazard ratio for primary outcome according to various subgroups using sotalol as a reference. The sotalol group was used as a reference group. *AADs* antiarrhythmic drugs, *mCCI* modified Charlson comorbidity index; IPTW was estimated using logistic regression with patient age, sex, CHA_2_DS_2_-VASc score, mCCI, number of AADs in baseline, baseline AADs, comorbidity, concomitant drugs, hospital type at index date, insurance type and index year. P-value was derived from Cox regression with IPTW in each subgroup. P-for-interaction was value for interaction term with treatment and each subgroups.

In subgroup analyses, the clinical benefits of dronedarone compared with those of sotalol were consistent across all the examined subgroups, except for the subgroup stratified by the CHA_2_DS_2_-VASc score (Fig. 3).

#### Sensitivity analyses for the primary outcome

Similar to the results observed during the entire study follow-up period, dronedarone use significantly reduced the risk of composite events with CV hospitalization and all-cause death when compared with sotalol use, both in the analyses restricting the follow-up period to 6 months and 12 months. Compared with sotalol users, the risk of the composite of CV hospitalization and all-cause death was significantly lower in dronedarone users with an HR of 0.60 (95% CI 0.50–0.71) at 6-month and HR of 0.63 (95% CI 0.54–0.74) at 12-month.

### Secondary outcomes

#### All-cause, CV, and non-CV hospitalization

Dronedarone use was associated with lower risks of all-cause hospitalization (HR 0.79, 95% CI 0.70–0.88) than sotalol use, as well as a lower risk of CV hospitalization (HR 0.62, 95% CI 0.53–0.72); however, there was no significant difference in the risk of non-CV hospitalization between the two groups (HR 1.05, 95% CI 0.91–1.22) (Fig. 2). Among the 10 predefined causes of CV hospitalization, the category of conduction disorders and arrhythmias was the most frequent cause of CV hospitalization across study groups. The risk of hospitalization owing to conduction disorders and arrhythmias, ischemic stroke and TIA, heart failure and hypertensive diseases were significantly lower in the dronedarone group than in the sotalol group (Supplementary Table S5 and Supplementary Fig. S4). We analyzed a more detailed reason for the hospitalization due to conduction disorders and arrhythmias. The dronedarone group showed significantly lower risks of hospitalization for I47.x (paroxysmal tachycardia) and I48.x (atrial fibrillation and flutter) than the sotalol group (Supplementary Table S6). In addition, the dronedarone group showed trends of lower risks of hospitalization due to I44.x (atrioventricular and left bundle branch block) and I49.x (other cardiac arrhythmias) than sotalol. For the risk of hospitalization for I45.x (other conduction disorder), two groups did not show any differences. When we reclassified the conduction disorders and arrhythmias into AF, bradycardia/sick sinus syndrome, atrioventricular block, and ventricular tachyarrhythmia, the dronedarone group was associated with lower risks of hospitalization due to AF and ventricular tachyarrhythmia than sotalol group (Supplementary Table S6). Dronedarone group tended to show lower risks of hospitalization for the risk of hospitalization for bradycardia/sick sinus syndrome and atrioventricular block than the sotalol group.

#### All-cause, CV, and non-CV death

The risks of all-cause death (HR 0.88, 95% CI 0.41–1.91), CV death (HR 0.23, 95% CI 0.04–1.25), and non-CV death (HR 1.33, 95% CI 0.52–3.44) did not significantly differ between the dronedarone and sotalol groups (Fig. 2).

#### Stroke and MI

Dronedarone use tended to be associated with a lower risk of stroke than sotalol use, presenting only a marginal and non-statistically significant association (HR 0.56, 95% CI 0.30–1.03) (Fig. 2). Additionally, the risk of MI did not differ between the two groups, (Fig. 2).

### Persistence and adherence

In the total population, 845 (18.0%) patients were persistent on the index AAD until the end of the follow-up period after the index date, whereas 3849 (82.0%) patients discontinued their index AAD. Approximately half of the patients (n = 2021, 52.5%) were switched to another AAD. The mean persistence time on the index AAD and the persistence rate were significantly higher in the dronedarone group than in the sotalol group (persistence time 368.0 vs. 270.2 days, persistence rate 21.0% vs. 12.0%: both p < 0.001). Of those patients who switched to another AAD from the index AAD, 3.8% in the dronedarone group switched to sotalol, whereas 10.5% in the sotalol group switched to dronedarone.

Adherence, assessed based on both a medication possession ratio (MPR) of ≥ 0.8 and the mean MPR, did not significantly differ between the two treatment groups. The detailed results are summarized in Supplementary Results, Supplementary Tables S7, and S8.

### Exploratory outcome: nonpharmacological rhythm control

The number of events, crude incidence, and weighted incidence of nonpharmacological rhythm control are summarized in Supplementary Table S4, and the weighted Kaplan–Meier curve is presented in Fig. 1. Among the total study population, 380 (8.1%) patients received nonpharmacological rhythm control (either electrical cardioversion [n = 263, 5.6%] or catheter ablation [n = 198, 4.2%]) during the study period. Dronedarone use was associated with a lower risk of nonpharmacological rhythm control than sotalol use (HR 0.63, 95% CI 0.51–0.77) (Fig. 2 and Supplementary Table S5).

### Hospitalization owing to potential safety signals including ALI, thyroid disorder, and ILD

No significant difference was observed in the risk of hospitalization owing to ALI (HR 1.71, 95% CI 0.41–7.19, p = 0.462). Although hospitalizations attributed to thyroid disease (n = 1) or ILD (n = 2) were observed in the dronedarone group only, the statistical difference between the two groups could not be examined.

## Discussion

To our knowledge, this is the first population-based observational study assessing the contemporary safety and effectiveness of dronedarone compared with an alternative antiarrhythmic agent with a similar indication (i.e., sotalol) in a real-world clinical setting in an Asian population. In the present study, dronedarone use revealed significantly lower risks of primary outcome defined as the composite of CV hospitalization and all-cause death, as well as a significantly lower need for nonpharmacological rhythm control, than sotalol in a large Asian population of AF patients. Conversely, there were no significant differences in the risks of all-cause mortality and non-CV hospitalization between groups. Furthermore, the dronedarone group presented a significantly lower risk of discontinuation than the sotalol group in real-world clinical practice. In subgroup analyses, our findings indicated that the benefits of dronedarone compared with sotalol were consistent across subgroups stratified based on baseline characteristics.

In terms of reduced risks of CV hospitalization, superior outcomes in the dronedarone group are consistent with the findings of previous clinical trials. In the ATHENA study, the risk of CV hospitalization was significantly lower in the dronedarone group than in the placebo group (HR 0.74, 95% CI 0.68–0.84)^8^. Furthermore, a significant reduction in the risk of stroke was observed with dronedarone when compared with the placebo in a post hoc analysis of the ATHENA study (HR 0.66, 95% CI 0.46–0.96)^24^. Regarding AF control, dronedarone has been associated with a significant reduction in AF recurrence in the EURIDIS/ADONIS trial^25^, demonstrating a 32% reduction in the risk of receiving electrical cardioversion when compared with placebo in a sub-analysis of ATHENA^26^. A mixed treatment meta-analysis of several previous clinical trials on the efficacy and safety of AADs, including dronedarone, sotalol, amiodarone, propafenone, and flecainide, has been performed^10^. According to this report, dronedarone presents better efficacy in preventing AF recurrence than the other AADs, with better safety than sotalol in terms of all-cause mortality. Moreover, dronedarone has revealed better safety than amiodarone regarding serious adverse events, demonstrating comparable results to other AADs in terms of proarrhythmic events^10^.

Recently, several real-world safety studies concerning dronedarone have presented consistent results with those of clinical trials^27^. In a Swedish nationwide cohort study, dronedarone users have demonstrated lower all-cause mortality than users of other AADs, including sotalol and amiodarone^11^. Reportedly, the risks of arrhythmic death, resuscitation, ventricular tachyarrhythmia, and new implantation of implantable cardioverter-defibrillator were significantly lower in the dronedarone group than in the sotalol group (HR 0.58, 95% CI 0.37–0.90)^28^. In a recent report from Germany, dronedarone has been associated with a lower risk of MI and stroke/TIA when compared with other AADs^29^. Furthermore, real-world data has demonstrated comparable safety profiles between dronedarone and other AADs in terms of incident liver diseases and drug-drug interactions with non-vitamin K antagonist oral anticoagulants^11,30,31^.

Both clinical trials and recent real-world evidence have proven that dronedarone has a reliable efficacy and safety profile. However, evidence in the Asian population, especially within real-world settings, is lacking. Our findings are consistent with those of previous reports in terms of reduced CV hospitalization^8,24,32^. Notably, the risks of CV hospitalization attributed to conduction disorders and arrhythmia (HR 0.59, 95% CI 0.49–0.70), ischemic stroke and TIA (HR 0.58, 95% CI 0.37–0.89), and heart failure (HR 0.46, 95% CI 0.22–0.95) were significantly lower in the dronedarone group than in the sotalol group. According to the risk of hospitalization for conduction disorders and arrhythmias, the dronedarone group was associated with lower risks of hospitalization for AF and ventricular tachyarrhythmia than the sotalol group. Dronedarone group tended to show lower risks of hospitalization for the risk of hospitalization for bradycardia/sick sinus syndrome and atrioventricular block than the sotalol group. These results could be carefully interpreted as dronedarone might have better effectiveness for AF control (reducing hospitalization for AF) and also have a less proarrhythmic effect than the sotalol group. Consistent with the previous studies, dronedarone might have a more favorable CV safety profile than sotalol in patients with AF in the comprehensive Cochrane review and the data from a real-world setting^27,28^. Furthermore, in the exploratory analysis, dronedarone use was associated with a lower need for nonpharmacological rhythm control than sotalol use (HR 0.63, 95% CI 0.51–0.77). Regarding the efficacy of AF rhythm control, dronedarone might provide a better option than sotalol.

In this study, both groups presented high discontinuation rates of index drug. The precise reason for discontinuation could not be analyzed owing to the inherent limitation of the claims database. However, we assumed that the lower discontinuation rate of dronedarone could be rationalized by the lower withdrawals due to adverse events or lower proarrhythmic effects when compared with sotalol. In the previously mentioned Cochrane review^27^, the relative risk (RR) for withdrawals owing to adverse events of dronedarone was 1.58 (95% CI 1.34–1.85), and that of sotalol was 1.95 (95% CI 1.23–3.11) when compared with a placebo or no treatment. Sotalol demonstrated a marginally higher RR for withdrawals than dronedarone owing to adverse events. The RR of dronedarone for proarrhythmia was 1.95 (95% CI 0.77–4.98), and that of sotalol was 3.55 (95% CI 2.16–5.83) when compared with a placebo or no treatment. Furthermore, the dronedarone group showed a significantly lower risk of non-pharmacological rhythm control than the sotalol group in this study, which could be another possible reason for the lower risk of drug discontinuation in the dronedarone group.

Concerning potential safety signals of interest, including ALI, thyroid disorder, and ILD, although the number of events was extremely low to identify any difference between the two treatment groups, dronedarone use did not appear to be associated with increased risks of potential safety signals in real-world settings. A recent Swedish study using the nationwide patient registry has reported that the risk of liver disease was not increased in the dronedarone group, with no death attributed to a liver disease diagnosis^11^. In a retrospective study from Germany, no documented cases of toxic liver disease were reported in patients prescribed dronedarone or other AADs^29^. Moreover, a post hoc analysis of the ATHENA study found no significant increase in the rate of thyroid or pulmonary disorders with dronedarone use^24,33^. Regarding the risk of death and potential safety signals, the findings of this study suggest that dronedarone might present a benign safety profile in real-world settings.

### Study limitations

Several limitations should be considered when interpreting our results. First, there was a possibility of miscoding and misclassifications that might have resulted in underestimation or overestimation of clinical outcomes. Second, death events were limited to in-hospital deaths owing to the nature of claims data. Therefore, the rate of all-cause death would be less observed in this study and the number of all-cause death events would be underestimated. Third, it was not possible to verify whether patients took the medicines as prescribed owing to the nature of claims data. Furthermore, we were unable to distinguish discontinuation owing to therapeutic needs, such as adverse events or insufficient therapeutic results, because the reasons for medication discontinuation were not reported in the national claims data. Fourth, in this analysis, we could not identify the exact reason or situation for receiving nonpharmacological rhythm control during follow-up. Therefore, nonpharmacological rhythm control was defined as an “exploratory outcome” for assuming the effectiveness of rhythm control indirectly. AF recurrence or AF burden could be better parameters to address a lower risk of CV hospitalization and all-cause death in the dronedarone group than the sotalol group. However, because of the inherent limitations of the claims database, we could not evaluate the AF recurrence or AF burden as clinical outcomes. Further investigations are crucial to demonstrate the comparative efficacy of dronedarone versus sotalol on AF recurrence or AF burden. Lastly, although we performed in-depth propensity score matching, the possibility of remaining bias and residual confounding factors cannot be ruled, out as this was a registry analysis.

## Conclusion

In this large-scale population of Asian patients with AF, dronedarone use was associated with lower risks of CV hospitalization, demonstrating a lower need for nonpharmacological rhythm control than sotalol use. Dronedarone might be an effective and safe treatment option for Asian patients with AF.

## Supplementary information

Supplementary Information.
