# Supplementary material for: Comparative clinical outcomes of dronedarone and sotalol in Asian patients with atrial fibrillation: a nationwide cohort study
Source: Sci Rep. 2020 Sep 30;10:16102. doi: 10.1038/s41598-020-73115-y (PMC7527331; doi:10.1038/s41598-020-73115-y)
Supplement: Supplementary file 1 — Supplementary Information. [file 41598_2020_73115_MOESM1_ESM.docx]

**Comparative clinical outcomes of dronedarone and sotalol in Asian patients with atrial fibrillation: A nationwide cohort study**

So-Ryoung Lee^1^, Eue-Keun Choi^1^, Ji-Hyun Kim^2^, Jung-Ae Kim^3^, Tae-Yeon Kwon^2^, Young Eun Lee^3^, Seil Oh^1^

^1^ Division of Cardiology, Department of Internal Medicine, Seoul National University Hospital, Seoul, Republic of Korea

^2^ Sanofi Korea, Seoul, Republic of Korea

^3^ Real World Insights, IQVIA Korea, Seoul, Republic of Korea

**Supplementary materials**

**I. Supplementary Methods**

**II. Supplementary Results**

**II. Supplementary Tables**

**IV. Supplementary Figures.**

**I. Supplementary Methods**

**Inclusion/exclusion criteria** **for the study population**

**Inclusion criteria**

Patients meeting all the following inclusion criteria were included:

1. Patients who received dronedarone or sotalol with no less than 7 days of supply during the index period (August 1, 2013, to December 31, 2016)
2. Patients who had at least 1 claim with diagnosis code of atrial fibrillation during 36-months prior to the index date (i.e. first prescription of dronedarone or sotalol)

Diagnosis codes for atrial fibrillation were attached in Online Table 1. The HIRA revised the 6^th^ version of Korea Classification of Disease code (KCD code) on January 01, 2016, in accordance with the International Classification of Disease-10 code (ICD-10 code). Therefore, the 6^th^ version of KCD code has been applied to the data from August 1, 2013, through December 31, 2015, and the 7^th^ version of KCD code has been applied to the data from January 1, 2016, through December 31, 2017.

**Exclusion criteria**

Patients meeting any of the following exclusion criteria were excluded:

1. Patients aged less 18 years as of the index date (< 18 years old)
2. Patients who had diagnosed with cancer and end-stage renal disease (ESRD) during the study period
3. Patients who received dronedarone or sotalol with longer than 7 days of supply during the baseline period
4. Patients who were diagnosed with contraindication of dronedarone or sotalol during the baseline period
   - Heart failure (ICD-10: I50.x)
   - Sick sinus syndrome or II- or III-degree atrioventricular (AV) block (ICD-10: I44.1, I44.2, I44.3, I49.5)
   - Bradycardia (ICD-10: R00.1)
   - Long QT syndrome (ICD-10: I49.8, I45.8)
   - Severe hepatic impairment (ICD-10: K72.x)
   - Pregnancy (ICD-10: O80.x, O81.x, O82.x, O83.x, O84.x, Z34.x)
   - Cardiogenic shock (ICD-10: R57.0)
   - Hypokalemia (ICD-10: E87.6)
   - Bronchial asthma (ICD-10: J45.x, J46.x)
5. Patients who were prescribed with the contraindicated medication of dronedarone or sotalol during the baseline period. The contraindicated medication was defined using the World Health Organization Anatomical Classification Code (WHO-ATC).
   - Strong CYP3A inhibitors
     - Ketoconazole (WHO-ATC: J02AB02)
     - Itraconazole (WHO-ATC: J02AC02)
     - Voriconazole (WHO-ATC: J02AC03)
     - Cyclosporine (WHO-ATC: L04AD01)
     - Telithromycin (WHO-ATC: J01FA15)
     - Clarithromycin (WHO-ATC: J01FA09)
     - Ritonavir (WHO-ATC: J05AE03)
   - Phenothiazine (WHO-ATC: J05AA, J05AB, J05AC)
   - Tricyclic antidepressants (WHO-ATC: N06AA)
   - Macrolide antibiotics (WHO-ATC: J01FA)
   - Class Ic and III antiarrhythmics combination
     - Class Ic, flecainide (WHO-ATC: C01BC04)
     - Class Ic, propafenone (WHO-ATC: C01BC03)
     - Class Ic, pilsicainide (WHO-ATC: C01BG)
     - Class III, dronedarone (WHO-ATC: C01BD07)
     - Class III, sotalol (WHO-ATC: C07AA07)
     - Class III, amiodarone (WHO-ATC: C01BD01)

Relevant diagnosis codes of contraindication and drug codes of contraindicated medication were identified from dronedarone prescribing information [1]. Relevant diagnosis codes, drug codes, and detailed operational definition for exclusion criteria were listed in Online Table 1.

**Definitions of covariates**

- Number of baseline AADs: number of AADs prescribed in the period of baseline
- Baseline AADs: AADs prescribed in the period of baseline
- Hospital type
  - Determined as the type of hospital prescribing the index AAD at the index date and categorized based on HIRA’s classification (i.e. tertiary hospital, general hospital, and general practitioner)
- Insurance type
  - Determined as a type of health insurance as of the index date (i.e. national health insurance or medical aid)
- Index year
  - Determined as the calendar year of the index date (i.e. 2013, 2014, 2015, 2016)

International Classification of Diseases (ICD) codes are used to identify comorbidities or clinical outcomes in a population-based study using administrative claims data. However, some concerns about the reliability of diagnosis coding with ICD-code have been raised due to inconsistencies and ambiguities of ICD-code [1]. Also, coding errors such as misspecification, re-sequencing, or substitution of diagnosis for the purpose of reimbursement or upcoding has also been addressed as an issue influencing the reliability of diagnostic codes [2]. Consequently, defining comorbidities using diagnostic codes has the potential for over-or under-estimation.

Several studies, hence, have been conducted to assess the reliability of diagnostic codes, and some of them reported favorable reliability in using ICD-code to identify comorbidities or clinical outcomes. Kim (2005), assessed the validity of diagnosing diabetes with ICD-10 codes (E10-E14) in Korean national health claims data and reported that the accuracy of identifying patients with diabetes was 87.2% (±3.4%) and 72.3% (±4.9%) for inpatient and outpatient claims respectively [3]. Similar results were also founded in studies assessing the accuracy of diagnostic codes for cardiovascular or cerebrovascular disease. Park et al. (2000) reported that the accuracy of diagnostic code for stroke was 83.4% for I63 and 71.4% for I64-I68 in Korean national health claims data [4]. Kim et al. (2011) reported that the accuracy of diagnosing acute myocardial infarction (AMI) using ICD-code of I21 was >70% and reliability was fair to good with the kappa value of 0.46 or 0.74 according to the World Health Organization criteria and European Society of Cardiology/American College of Cardiology (ESC/ACC), respectively [5]. Park et al. (2019), evaluated the validation of diagnostic codes of major clinical outcomes in Koran national health claims data, and demonstrated that diagnosing AMI and gastrointestinal bleeding (GIB) using ICD-10 code were reliable with the positive predictive value (PPV) of 92% for AMI and 82% for GIB based on primary diagnostic codes at discharge. For stroke and intracranial hemorrhage (ICH), the primary discharge diagnostic codes showed the PPV of 90.5% and 71.5%, respectively [6]. Similarly, Park et al. (2003) reported that the overall PPV of all diagnoses was about 70% in Korean national health claims data [7]. Also, Kim (2016) suggested that there was no significant difference in predictability in health outcome studies using all diagnoses of both in-patients and out-patients to define comorbidity compared to other approaches [8].

**Definitions of drug persistence and adherence**

**Persistence**

Persistence was measured by the proportion of patients persistent on the index AAD. Patients were considered as persistent if they renewed their previous index AAD prescription within a defined grace period from the ending of the previous index AAD prescription. The grace period was defined as a period between two consecutive prescriptions from the end date of the previous prescription to the start date of the following prescription. This study used the grace period of 30-days. For example, a patient was persistent on the index AAD if the patient renewed the previous index AAD prescription within 30-days from the ending of the previous index AAD prescription. The continuation and discontinuation were categorized according to the following definition, and the discontinuation was further classified as stop, restart, or switch.

- Continuation: Continuously renew the index AAD within a defined grace period of 30-days
- Discontinuation: Fail to renew the index AAD within a defined grace period of 30-days
  - Stop: No further subsequent prescription for index AAD observed until the end of the study period from the ending of the previous index AAD
  - Restart: Renew the index AAD with a gap in therapy > 30days from the ending of the previous index AAD
  - Switch: Refill a prescription with a different AAD within a defined grace period of 30-days or with a gap of >30days from the ending of the previous index AAD
  - Add-on: Add other AADs except for index AAD in subsequent prescription

AADs considered in this study were dronedarone, sotalol, amiodarone, flecainide, propafenone, and pilsicainide. Therefore, only pre-defined six AADs were considered to assess a switch. For example, it was assessed as a stop if a patient received a prescription of other class AADs such as verapamil, propranolol from the ending of the previous prescription for the index AAD.

**Adherence**

Adherence was measured by the medication possession ratio (MPR). MPR was calculated as the sum of days’ supply of the index AAD divided by the total exposure period (follow-up period) which was defined as the duration from initiation of the index AAD to discontinuation. Patients with MPR≥ 80% were considered adherent and MPR<80% was non-adherent.

Description methods of drug persistence and adherence

Persistence was presented as the number of patients and proportion (%) of patients persistent on their index AAD. Persistence time on the index AAD was also presented as mean, standard deviation, minimum, maximum, median, 25^th^ and 75^th^ percentile. The discontinuation was further classified either as “stop”, “switch”, “restart”, or “add-on”, and it was presented as the number of patients and proportion (%) of patients for each classification. MPR was presented as mean, standard deviation, minimum, maximum, median, 25^th^ and 75^th^ percentile.

**References**

[1] Sanofi-Aventis Korea. Multaq tab. (dronedarone hydrochloride) [package insert]. KFDA. website. http://products.sanofi.co.kr/KR_Multaq%20tab_2017-08-10.pdf revised August 2017. Accessed September 12, 2018.

[2] Surjan, G. Questions on validity of international classification of diseases-coded diagnosis. *Int J Med Inform.* ***54****, 77-95 (*1999).

[3] Hsia, D. C., Ahern, C. A., Ritchie, B. P., Moscoe, L. M. & Krushat, W. M. Medicare reimbursement accuracy under the prospective payment system, 1985 to 1988. *JAMA*. **268**, 896-899 (1992).

[4] Kim, J. W. Analysis in the incidence and hospital use of Diabetes using medical insurance claim data. *Diabetes in Korea.* 42-55 (2005).

[5] Park, J. K., et al. The accuracy of ICD codes for cerebrovascular diseases in medical insurance claims. *J Prev Med Public Health.* **33**, 76-82 (2000).

[6] Kimm, H. J., Yun, J. E., Lee, S. H., Jang, Y. S. & Jee, S. H. Validity of the diagnosis of acute myocardial infarction in Korean national medical health insurance claims data: The Korean Heart Study [1]. *Korean Circ J.* **42**, 10-15 (2012)..

[7] Park, J. S., et al. Validation of diagnostic codes of major clinical outcomes in a National Health Insurance database. Int J Arrhythm. **20**; 10.1186/s42444-019-0005-0 (2019).

[8] Park, B. J., Sung, J. H., Park, K. D., Seo, S. W. & Kim, S. W. Report of the evaluation for validity of discharged diagnoses in Korean health insurance database. *Seoul National University*, 19-52 (2003).

[9] Kim, K. H. Comobidity adjustment in health insurance claim database. *Health Policy and Management,* **26**, 71-78 (2016).

**II. Supplementary Results**

**Persistence**

The persistence rate and persistent time on the index AAD are summarized in Supplementary Table S7.

Out of 4,694 patients enrolled in this study, 845 (18.0%) patients were persistent on the index AAD until the end of the follow-up period after the index date and the mean persistent time on the index AAD was 335.2 days. Among 3,849 (82.0%) patients with discontinuation of their index AAD, 1,073 (27.9%) patients stopped AAD treatment, 2,021 (52.5%) patients switched to another AAD, 654 (17.0%) patients restarted their index AAD with >30-days gap, and 101 (2.2%) patients received additional another AAD with ≥1 day overlap. For those who switched their index AAD to other AAD, Amiodarone (38.1%) was the most frequently selected AAD followed by Flecainide (26.4%) and Propafenone (19.6%). Further, 3.8% of switchers in dronedarone group switched to sotalol whereas 10.5% of switchers in sotalol group switched to dronedarone.

Dronedarone group (21.0%) showed significantly higher persistence rate than sotalol group (12.0%) (p<0.001). For those who discontinued their index AAD, the proportion of switcher (i.e. switched the index AAD to other AAD) was significantly lower in dronedarone group (50.0%) than in sotalol group (56.9%) (p<0.001). Although proportions of stopper (i.e. stopped AAD treatment) and restarter (i.e. restarted the index AAD with >30-days gap) were higher in dronedarone group (stopper 29.0%, restarter 18.1%) than in sotalol group (stopper 25.8%, restarter 15.0%) but no significant difference was observed between both groups.

The mean persistent time on the index AAD was significantly longer in dronedarone group (368.0 days) than sotalol group (270.2 days) (p<0.001). For those who were persistent on the index AAD until the end of follow-up period, the mean persistent period on the index AAD was significantly longer in dronedarone group (888.0 days) than sotalol group (776.7 days) (p<0.001). On the other hand, the mean persistent time in both stoppers and switchers was not significantly different between the study groups. In contrast, the mean persistent time among restarters was significantly longer in dronedarone group (359.2 days) compared to that in sotalol group (270.2 days) (p<0.001).

**Adherence**

The adherence rate and proportion of adherent are summarized in Supplementary Table S8.

Out of 4,652 patients enrolled in this study, 99.1% patients was adherent with MPR ≥0.8 and the mean MPR was 1.03. The proportion of adherent and mean MPR were not significantly different between dronedarone and sotalol group. For those who continued their index AAD until the end of follow-up period, the mean MPR was significantly lower in dronedarone group (1.09) compared to that in sotalol group (1.13) (p=0.002). Moreover, the mean MPR was significantly higher among switchers and restarters in dronedarone group compared to that observed in sotalol group.

**III. Supplementary Tables**

**Supplementary Table S1. Relevant diagnosis codes, drug codes, and detailed operational definition for inclusion/exclusion criteria**

| Criteria | Class | Code | Detailed operational definition |
| --- | --- | --- | --- |
| **Inclusion criteria (applied to the data before January 2016)** | |  |  |
| Atrial fibrillation | Diagnosis | I48.0 | Admission ≥1 or outpatient department visit ≥2 with defined diagnosis code as primary-6^th^ diagnosis |
| Atrial flutter | Diagnosis | I48.1 |  |
| **Inclusion criteria (applied to the data after January 2016)** | |  |  |
| Paroxysmal atrial fibrillation | Diagnosis | I48.0 | Admission ≥1 or outpatient department visit ≥2 with defined diagnosis code as primary-6^th^ diagnosis |
| Persistent atrial fibrillation | Diagnosis | I48.1 |  |
| Chronic atrial fibrillation | Diagnosis | I48.2 |  |
| Typical atrial flutter | Diagnosis | I48.3 |  |
| Atypical atrial flutter | Diagnosis | I48.4 |  |
| Atrial fibrillation and atrial flutter, unspecified | Diagnosis | I48.9 |  |
| **Exclusion criteria (applied to the data before January 2016)** | |  |  |
| Malignant neoplasms | Diagnosis | C00.x-C97.x | Admission or outpatient department visit ≥1 with defined diagnosis code as primary-6^th^ diagnosis |
| Hemodialysis | Procedure | O7020 | Dialysis (hemodialysis or peritoneal dialysis) ≥2 |
| Continuous Venovenous or Arteriovenous Hemodialysis | Procedure | O7035 |  |
| Continuous Venovenous Hemodialysis | Procedure | O7031 |  |
|  | Procedure | O7032 |  |
| Continuous Arteriovenous Hemodialysis | Procedure | O7033 |  |
|  | Procedure | O7034 |  |
| Acute Peritoneal Dialysis | Procedure | O7061 |  |
|  | Procedure | O7062 |  |
| Break-in for Chronic Peritoneal Dialysis | Procedure | O7074 |  |
| **Exclusion criteria (applied to the data after January 2016)** | |  |  |
| Malignant neoplasms | Diagnosis | C00.x-C97.x | Admission or outpatient department visit ≥1 with defined diagnosis code as primary-6^th^ diagnosis |
| Hemodialysis | Procedure | O7020 | Dialysis (hemodialysis or peritoneal dialysis) ≥2 |
| Continuous Venovenous Hemodialysis | Procedure | O7031 |  |
|  | Procedure | O7032 |  |
| Continuous Arteriovenous Hemodialysis | Procedure | O7033 |  |
|  | Procedure | O7034 |  |
| Continuous Venovenous or Arteriovenous Hemodialysis | Procedure | O7035 |  |
| Acute Peritoneal Dialysis | Procedure | O7061 |  |
|  | Procedure | O7062 |  |
| Break-in for Chronic Peritoneal Dialysis | Procedure | O7074 |  |
| **Contraindication of Dronedarone** |  |  |  |
| NYHA Class IV heart failure or NYHA Class II - III heart failure with a recent decompensation requiring hospitalization or referral to a specialized heart failure clinic |  |  |  |
| Heart failure | Diagnosis | I50.x | Admission ≥1 with defined diagnosis code as primary diagnosis during baseline period |
| Second- or third-degree atrioventricular (AV) block or sick sinus syndrome (except when used in conjunction with a functioning pacemaker) |  |  |  |
| Atrioventricular block, second degree | Diagnosis | I44.1 | Admission or outpatient department visit ≥1 with defined diagnosis as any diagnosis during baseline period |
| Atrioventricular block, type I and II | Diagnosis | I44.1 |  |
| Mobitz block, type I and II | Diagnosis | I44.1 |  |
| Second-degree block, type I and II | Diagnosis | I44.1 |  |
| Wenkebach’s block | Diagnosis | I44.1 |  |
| Atrioventricular block, complete | Diagnosis | I44.2 |  |
| Complete heart block NOS | Diagnosis | I44.2 |  |
| Third-degree block | Diagnosis | I44.2 |  |
| Other and unspecified atrioventricular block | Diagnosis | I44.3 |  |
| Atrioventricular block NOS | Diagnosis | I44.3 |  |
| Sick sinus syndrome | Diagnosis | I49.5 |  |
| Tachycardia-bradycardia syndrome | Diagnosis | I49.5 |  |
| Bradycardia <50 bpm |  |  |  |
| Bradycardia, unspecified | Diagnosis | R00.1 | Admission or outpatient department visit ≥1 with defined diagnosis code as primary-6^th^ diagnosis during baseline period |
| QTc Bazett interval ≥500 ms or PR interval >280 ms |  |  |  |
| Long QT syndrome (after January 2016) | Diagnosis | I49.8 | Admission or outpatient department visit ≥1 with defined diagnosis code as primary-6^th^ diagnosis during baseline period |
| Long QT syndrome (before January 2016) | Diagnosis | I45.8 |  |
| Severe hepatic impairment |  |  |  |
| Hepatic failure, NEC | Diagnosis | K72.x | Admission ≥1 with defined diagnosis code as primary diagnosis during baseline period |
| Hepatic coma NOS | Diagnosis | K72.x |  |
| Hepatic encephalopathy NOS | Diagnosis | K72.x |  |
| Fulminant hepatitis with hepatic failure NEC | Diagnosis | K72.x |  |
| Malignant hepatitis with hepatic failure NEC | Diagnosis | K72.x |  |
| Liver (cell) necrosis with hepatic failure | Diagnosis | K72.x |  |
| Yellow liver atrophy or dystrophy | Diagnosis | K72.x |  |
| Pregnancy, Nursing mothers |  |  |  |
| Delivery(O80-O84) | Diagnosis | O80-O84 | Admission or outpatient department visit ≥1 with defined diagnosis code as primary-6^th^ diagnosis during baseline period |
| Supervision of normal pregnancy | Diagnosis | Z34.x |  |
| **Contraindication of Sotalol** |  |  |  |
| Uncontrolled heart failure |  |  |  |
| Heart failure | Diagnosis | I50.x | Admission ≥1 admission with defined diagnosis code as primary diagnosis during baseline period |
| Sick sinus syndrome or second and third degree AV block (unless a functioning pacemaker is present) |  |  |  |
| Atrioventricular block, second degree | Diagnosis | I44.1 | Admission or outpatient department visit ≥1 with defined diagnosis code as any diagnosis during baseline period |
| Atrioventricular block, type I and II | Diagnosis | I44.1 |  |
| Mobitz block, type I and II | Diagnosis | I44.1 |  |
| Second-degree block, type I and II | Diagnosis | I44.1 |  |
| Wenkebach’s block | Diagnosis | I44.1 |  |
| Atrioventricular block, complete | Diagnosis | I44.2 |  |
| Complete heart block NOS | Diagnosis | I44.2 |  |
| Third-degree block | Diagnosis | I44.2 |  |
| Other and unspecified atrioventricular block | Diagnosis | I44.3 |  |
| Atrioventricular block NOS | Diagnosis | I44.3 |  |
| Sick sinus syndrome | Diagnosis | I49.5 |  |
| Tachycardia-bradycardia syndrome | Diagnosis | I49.5 |  |
| Sinus bradycardia (<50 bpm during waking hours), |  |  |  |
| Bradycardia, unspecified | Diagnosis | R00.1 | Admission or outpatient department visit ≥1 with defined diagnosis code as primary-6^th^ diagnosis during baseline period |
| Congenital or acquired long QT syndromes, baseline QT interval >450 msec, |  |  |  |
| Long QT syndrome (after January 2016) | Diagnosis | I49.8 | Admission or outpatient department visit ≥1 with defined diagnosis code as primary-6^th^ diagnosis during baseline period |
| Long QT syndrome (before January 2016) | Diagnosis | I45.8 |  |
| Cardiogenic shock |  |  |  |
| Cardiogenic shock | Diagnosis | R57.0 | Admission ≥1 with defined diagnosis code as primary diagnosis during baseline period |
| Hypokalemia (<4 meq/L) |  |  |  |
| Potassium[K] deficiency | Diagnosis | E87.6 | Admission or outpatient department visit ≥1 with defined diagnosis code as primary-6^th^ diagnosis during baseline period |
| Bronchial asthma and previous evidence of hypersensitivity to sotalol |  |  |  |
| Asthma | Diagnosis | J45.x | Admission or outpatient department visit ≥1 with defined diagnosis code as primary-6^th^ diagnosis during baseline period |
| Status asthmaticus | Diagnosis | J46.x |  |
| **Contraindicated medication of Dronedarone** |  |  |  |
| Concomitant use of strong CYP 3A inhibitors, such as ketoconazole, itraconazole ole, voriconazole, cyclosporine, telithromycin, clarithromycin, nefazodone, and ritonavir |  |  |  |
| Ketoconazole (WHO-ATC: J02AB02) |  |  |  |
| Ketoconazole 200mg | Drug | 179601ATB | Admission or outpatient department visit ≥1 with prescription of defined drug during baseline or follow-up period |
| Itraconazole (J02AC02) |  |  |  |
| Itraconazole 0.25g(10mg/ml) | Drug | 179130BIJ | Admission or outpatient department visit ≥1 with prescription of defined drug during baseline or follow-up period |
| Itraconazole 1.5g(10mg/ml) | Drug | 179131ALQ |  |
| Itraconazole 100mg | Drug | 179101ACH |  |
| Itraconazole 100mg | Drug | 179101ATB |  |
| Itraconazole 10mg | Drug | 179102ALQ |  |
| Itraconazole 200mg | Drug | 179104ATB |  |
| Itraconazole 250mg | Drug | 179103BIJ |  |
| Voriconazole (J02AC03) |  |  |  |
| Voriconazole 200mg | Drug | 456501ATB | Admission or outpatient department visit ≥1 with prescription of defined drug during baseline or follow-up period |
| Voriconazole 200mg | Drug | 456501BIJ |  |
| Cyclosporine (L04AD01) |  |  |  |
| Cyclosporine 0.25g(50mg/ml) | Drug | 139230BIJ | Admission or outpatient department visit ≥1 with prescription of defined drug during baseline or follow-up period |
| Cyclosporine 100mg | Drug | 139201ACS |  |
| Cyclosporine 250mg | Drug | 139202BIJ |  |
| Cyclosporine 25mg | Drug | 139204ACS |  |
| Microemulsion Cyclosporine (L04AD01) |  |  |  |
| Microemulsion Cyclosporine 100mg | Drug | 194701ACS | Admission or outpatient department visit ≥1 with prescription of defined drug during baseline or follow-up period |
| Microemulsion Cyclosporine 100mg | Drug | 194701ALQ |  |
| Microemulsion Cyclosporine 10g(0.1g/ml) | Drug | 194731ALQ |  |
| Microemulsion Cyclosporine 25mg | Drug | 194702ACS |  |
| Microemulsion Cyclosporine 5g(0.1g/ml) | Drug | 194730ALQ |  |
| Telithromycin (J01FA15) |  |  |  |
| Telithromycin 400mg | Drug | 455901ATB | Admission or outpatient department visit ≥1 with prescription of defined drug during baseline or follow-up period |
| Clarithromycin (J01FA09) |  |  |  |
| Clarithromycin 0.125g(25mg/ml) | Drug | 134932ASY | Admission or outpatient department visit ≥1 with prescription of defined drug during baseline or follow-up period |
| Clarithromycin 0.75g(25mg/ml) | Drug | 134933ASY |  |
| Clarithromycin 1.5g(25mg/ml) | Drug | 134934ASY |  |
| Clarithromycin 11.25g(25mg/ml) | Drug | 134942ASY |  |
| Clarithromycin 2.5g(25mg/ml) | Drug | 134935ASY |  |
| Clarithromycin 2.5g(50mg/ml) | Drug | 134936ASY |  |
| Clarithromycin 250mg | Drug | 134901ATB |  |
| Clarithromycin 25mg | Drug | 134903ASY |  |
| Clarithromycin 25mg(25mg/ml) | Drug | 134930ASY |  |
| Clarithromycin 3.5g(50mg/ml) | Drug | 134937ASY |  |
| Clarithromycin 3.75g(25mg/ml) | Drug | 134938ASY |  |
| Clarithromycin 500mg | Drug | 134902BIJ |  |
| Clarithromycin 500mg | Drug | 134904ATB |  |
| Clarithromycin 500mg | Drug | 134904ATR |  |
| Clarithromycin 50mg | Drug | 134905ASY |  |
| Clarithromycin 50mg(50mg/ml) | Drug | 134931ASY |  |
| Clarithromycin 5g(50mg/ml) | Drug | 134939ASY |  |
| Clarithromycin 7.5g(25mg/ml) | Drug | 134941ASY |  |
| Clarithromycin 7g(50mg/ml) | Drug | 134940ASY |  |
| Nefazodone (N06AX06) |  |  |  |
| na | na | na | Admission or outpatient department visit ≥1 with prescription of defined drug during baseline or follow-up period |
| Ritonavir (J05AE03) |  |  |  |
| Ritonavir 100mg | Drug | 224401ACH | Admission or outpatient department visit ≥1 with prescription of defined drug during baseline or follow-up period |
| Ritonavir 100mg | Drug | 224401ACS |  |
| Ritonavir 100mg | Drug | 224401ATB |  |
| Ritonavir 19.2g(80mg/ml) | Drug | 224430ALQ |  |
| Ritonavir 80mg | Drug | 224402ALQ |  |
| Concomitant use of drugs or herbal products that prolong the QT interval and might increase the risk of Torsade de Pointes, such as phenothiazine anti-psychotics, tricyclic antidepressants, certain oral macrolide antibiotics, and Class I and III antiarrhythmics |  |  |  |
| Chlorpromazine (N05AA) |  |  |  |
| Chlorpromazine HCL 100mg | Drug | 131901ATB | Admission or outpatient department visit ≥1 with prescription of defined drug during baseline or follow-up period |
| Chlorpromazine HCL 200mg | Drug | 131905ATB |  |
| Chlorpromazine HCL 50mg | Drug | 131908ATB |  |
| Levomepromazine Maleate (N05AA) |  |  |  |
| Levomepromazine Maleate 100mg | Drug | 183301ATB | Admission or outpatient department visit ≥1 with prescription of defined drug during baseline or follow-up period |
| Levomepromazine Maleate 25mg | Drug | 183302ATB |  |
| Levomepromazine Maleate 50mg | Drug | 183303ATB |  |
| Perphenazine (N05AB) |  |  |  |
| Perphenazine 4mg | Drug | 211401ATB | Admission or outpatient department visit ≥1 with prescription of defined drug during baseline or follow-up period |
| Amitriptyline (N06AA) |  |  |  |
| Amitriptyline HCL 10mg | Drug | 107501ATB | Admission or outpatient department visit ≥1 with prescription of defined drug during baseline or follow-up period |
| Amitriptyline HCL 25mg | Drug | 107502ATB |  |
| Amitriptyline HCL 5mg | Drug | 107504ATB |  |
| Amoxapine (N06AA) |  |  |  |
| Amoxapine 100mg | Drug | 108001ATB | Admission or outpatient department visit ≥1 with prescription of defined drug during baseline or follow-up period |
| Amoxapine 50mg | Drug | 108002ATB |  |
| Clomipramine (N06AA) |  |  |  |
| Clomipramine HCL 10mg | Drug | 136301ACH | Admission or outpatient department visit ≥1 with prescription of defined drug during baseline or follow-up period |
| Clomipramine HCL 25mg | Drug | 136302ACH |  |
| Dothiepin (N06AA) |  |  |  |
| Dothiepin HCL 25mg | Drug | 148901ACH | Admission or outpatient department visit ≥1 with prescription of defined drug during baseline or follow-up period |
| Doxepin (N06AA) |  |  |  |
| Doxepin HCL (as Doxepin 3mg) | Drug | 149203ATB | Admission or outpatient department visit ≥1 with prescription of defined drug during baseline or follow-up period |
| Doxepin HCL (as Doxepin 6mg) | Drug | 149204ATB |  |
| Imipramine (N06AA) |  |  |  |
| Imipramine HCL 25mg | Drug | 173701ATB | Admission or outpatient department visit ≥1 with prescription of defined drug during baseline or follow-up period |
| Nortryptiline (N06AA) |  |  |  |
| Nortryptiline HCL 11.4mg | Drug | 203401ATB | Admission or outpatient department visit ≥1 with prescription of defined drug during baseline or follow-up period |
| Nortryptiline HCL 28.5mg | Drug | 203402ATB |  |
| Azithromycin (J01FA) |  |  |  |
| Azithromycin 0.6g(40mg/ml) | Drug | 112732ASY | Admission or outpatient department visit ≥1 with prescription of defined drug during baseline or follow-up period |
| Azithromycin 0.9gG(40mg/ml) | Drug | 112733ASY |  |
| Azithromycin 250mg | Drug | 112701ATB |  |
| Azithromycin 40mg | Drug | 112702ASY |  |
| Azithromycin 40mg(40mg/ml) | Drug | 112731ASY |  |
| Azithromycin 500mg | Drug | 112705ATB |  |
| Azithromycin Dihydrate (J01FA) |  |  |  |
| Azithromycin Dihydrate 524.1mg | Drug | 439901BIJ | Admission or outpatient department visit ≥1 with prescription of defined drug during baseline or follow-up period |
| Azithromycin Hydrate (J01FA) |  |  |  |
| Azithromycin Hydrate (as Azithromycin 0.5g) | Drug | 112734BIJ | Admission or outpatient department visit ≥1 with prescription of defined drug during baseline or follow-up period |
| Erythromycin Estolate (J01FA) |  |  |  |
| Erythromycin Estolate 1.5g(25mg/ml) | Drug | 153530ASY | Admission or outpatient department visit ≥1 with prescription of defined drug during baseline or follow-up period |
| Erythromycin Estolate 12.5g(25mg/ml) | Drug | 153531ASY |  |
| Erythromycin Estolate 250mg | Drug | 153501ACH |  |
| Erythromycin Estolate 25g(25mg/ml) | Drug | 153532ASY |  |
| Erythromycin Estolate 25mg | Drug | 153502ASY |  |
| Erythromycin Propionate (J01FA) |  |  |  |
| Erythromycin Propionate 500mg | Drug | 153602ATB | Admission or outpatient department visit ≥1 with prescription of defined drug during baseline or follow-up period |
| Erythromycin (J01FA) |  |  |  |
| Erythromycin(Enteric Coated 430mg) 250mg | Drug | 154001ACH | Admission or outpatient department visit ≥1 with prescription of defined drug during baseline or follow-up period |
| Josamycin (J01FA) |  |  |  |
| Josamycin 200mg | Drug | 179201ATB | Admission or outpatient department visit ≥1 with prescription of defined drug during baseline or follow-up period |
| Midecamycin Acetate (J01FA) |  |  |  |
| Midecamycin Acetate 0.2g(0.2g/g) | Drug | 195530ASY | Admission or outpatient department visit ≥1 with prescription of defined drug during baseline or follow-up period |
| Midecamycin Acetate 200mg | Drug | 195501ASY |  |
| Midecamycin Acetate 200mg | Drug | 195501ATB |  |
| Midecamycin Acetate 2g(0.2g/g) | Drug | 195531ASY |  |
| Midecamycin Acetate 60g(0.2g/g) | Drug | 195532ASY |  |
| Roxithromycin (J01FA) |  |  |  |
| Roxithromycin 0.25g(50mg/g) | Drug | 225330AGN | Admission or outpatient department visit ≥1 with prescription of defined drug during baseline or follow-up period |
| Roxithromycin 10g(50mg/g) | Drug | 225336AGN |  |
| Roxithromycin 10mg | Drug | 225304ASS |  |
| Roxithromycin 150mg | Drug | 225301ATB |  |
| Roxithromycin 15g(50mg/g) | Drug | 225337AGN |  |
| Roxithromycin 2.5g(50mg/g) | Drug | 225331AGN |  |
| Roxithromycin 50mg | Drug | 225302AGN |  |
| Roxithromycin 50mg | Drug | 225302ASS |  |
| Roxithromycin 50mg | Drug | 225302ATB |  |
| Roxithromycin 5g(10mg/ml) | Drug | 225332ASS |  |
| Roxithromycin 5g(50mg/g) | Drug | 225333AGN |  |
| Roxithromycin 6g(50mg/g) | Drug | 225334AGN |  |
| Roxithromycin 7.5g(50mg/g) | Drug | 225335AGN |  |
| Flecainide (C01BC) |  |  |  |
| Flecainide 150mg | Drug | 501902BIJ | Admission or outpatient department visit ≥1 with prescription of defined drug during baseline or follow-up period |
| Flecainide 50mg | Drug | 501901BIJ |  |
| Flecainide Acetate (C01BC) |  |  |  |
| Flecainide Acetate 150mg(10mg/ml) | Drug | 159331BIJ | Admission or outpatient department visit ≥1 with prescription of combination of class 1 and class3 antiarrhythmics with ≥90 days of supply during baseline or follow-up period |
| Flecainide Acetate 50mg | Drug | 159302ATB |  |
| Flecainide Acetate 50mg(10mg/ml) | Drug | 159330BIJ |  |
| Pilsicainide (C01BG) |  |  |  |
| Pilsicainide HCL 25mg | Drug | 502101ACH | Admission or outpatient department visit ≥1 with prescription of combination of class 1 and class3 antiarrhythmics with ≥90 days of supply during baseline or follow-up period |
| Pilsicainide HCL 50mg | Drug | 502102ACH |  |
| Propafenone (C01BC) |  |  |  |
| Propafenone HCL 150mg | Drug | 219501ATB | Admission or outpatient department visit ≥1 with prescription of combination of class 1 and class3 antiarrhythmics with ≥90 days of supply during baseline or follow-up period |
| Propafenone HCL 225mg | Drug | 219503ACR |  |
| Propafenone HCL 300mg | Drug | 219502ATB |  |
| Propafenone HCL 325mg | Drug | 219504ACR |  |
| Propafenone HCL 425mg | Drug | 219505ACR |  |
| Amiodarone (C01BD) |  |  |  |
| Amiodarone HCL 150mg | Drug | 107402BIJ | Admission or outpatient department visit ≥1 with prescription of combination of class 1 and class3 antiarrhythmics with ≥90 days of supply during baseline or follow-up period |
| Amiodarone HCL 200mg | Drug | 107401ATB |  |
| Amiodarone HCL 150mg(50mg/ml) | Drug | 107430BIJ |  |
| Dronedarone (C01BD) |  |  |  |
| Dronedarone 400mg | Drug | 597401ATB | Admission or outpatient department visit ≥1 with prescription of combination of class 1 and class3 antiarrhythmics with ≥90 days of supply during baseline or follow-up period |
| Sotalol (C07AA) |  |  |  |
| Sotalol HCL 160mg | Drug | 230401ATB | Admission or outpatient department visit ≥1 with prescription of combination of class 1 and class3 antiarrhythmics with ≥90 days of supply during baseline or follow-up period |
| Sotalol HCL 40mg | Drug | 230402ATB |  |

The diagnosis was coded according to Korea Classification of Disease code (KCD code) modification of the International Classification of Disease-10 code (ICD-10 code). The 6th version of KCD code was applied to the data from August 1,2013 to December 31, 2015 and the 7th version of KCD code was applied to the data from January 1, 2016 to December 31, 2017 since the 6^th^ version of KCD code has been revised at January 01, 2016. The drug was coded according to Health Insurance Review and Assessment’s molecule code. The procedure was coded according to Health Insurance Review and Assessment reimbursed code.

**Supplementary Table S2. Operational definitions for comorbidities, CHA_2_DS_2_-VASc score, and modified CCI**

| Operational definition | Diagnosis code | Detailed operational definition |
| --- | --- | --- |
| **CHA2DS2 -VASc score** |  |  |
| Congestive heart failure (1 point) | I50.0 | Admission or outpatient department visit ≥1 with defined diagnosis code as primary-6^th^ diagnosis |
| Hypertension (1 point) | I10.x–I13.x, I15.x |  |
| Age ≥ 75 years (2 point) | n/a | Age at index date |
| Age 65-74 years (1 point) | n/a |  |
| Sex category (female) (1 point) | n/a | Sex at index date |
| Diabetes mellitus (1 point) | E10.x–E14.x | Admission or outpatient department visit ≥1 with defined diagnosis code as primary-6^th^ diagnosis |
| Stroke/TIA/TE (2 point) | I63.x, I64.x, I74.x, G45.x |  |
| Vascular disease (prior MI, PAD or aortic plaque) (1 point) | I21.x, I22.x, I23.x, I70.x |  |
| **Modified Charlson comorbidity index** |  |  |
| Congestive heart failure (2 point) | I09.9, I11.0, I13.0, I13.2, I25.5, I42.0, I42.5-I42.9, I43.x, I50.x, P29.0 | Admission or outpatient department visit ≥1 with defined diagnosis code as primary-6^th^ diagnosis |
| Dementia (2 point) | F00.x-F03.x, F05.1, G31.1 |  |
| Chronic pulmonary disease (1 point) | I27.8, I27.9, J40.x-J47.x, J601.x-J67.x, J68.4, J70.1, J70.3 |  |
| Rheumatologic disease (1 point) | M05.x, M06.x, M31.5, M32.x-M34.x, M35.1, M35.3, M36.0 |  |
| Mild liver disease (2 point) | B18.x, K70.0-K70.3, K70.9, K71.3-K71.5, K71.7, K73.x, K74.x, K76.0, K76.2-K76.4, K76.8, K76.9, Z94.4 |  |
| Diabetes with chronic complications (1 point) | E10.2-E10.5, E10.7, E11.2-E11.5, E11.7, E12.2-E12.5, E12.7, E13.2-E13.5, E13.7, E14.2-E14.5, E14.7 |  |
| Hemiplegia or paraplegia (2 point) | G04.1, G11.4, G80.1, G80.2, G81.x, G82.x, G83.0-G83.4, G83.9 |  |
| Renal disease (1 point) | I12.0, I13.1, N03.2-N03.7, N05.2-N05.7, N18.x, N19.x, N25.0, Z49.0-Z49.2, Z94.0, Z99.2 |  |
| Any malignancy, including lymphoma and leukemia (2 point) | C00.x-C26.x, C30.x-C34.x, C37.x-C41.x, C43.x, C45.x-C58.x, C60.x-C76.x, C81.x-C85.x, C88.x, C90.x-C97.x |  |
| Moderate or severe liver disease (4 point) | I85.0, I85.9, I86.4, I98.2, K70.4, K71.1, K72.1, K72.9, K76.5, K76.6, K76.7 |  |
| Metastatic solid tumor (6 point) | C77.x-C80.x |  |
| HIV (4 point) | B20.x-B22.x, B24.x |  |
| **Comorbidities** |  |  |
| Hypertension | I10-I15 | Admission ≥1 or outpatient department visit ≥2 with defined diagnosis code as primary-6^th^ diagnosis |
| Diabetes mellitus (DM) | E11-E14 |  |
| Congestive heart failure (CHF) | I50 | Admission or outpatient department visit ≥1 with defined diagnosis code as primary-6^th^ diagnosis |
| Ischemic heart disease (IHD) | I20-I25 | Admission ≥1 or outpatient department visit ≥2 with defined diagnosis code as primary-6^th^ diagnosis |
| Myocardial infarction (MI) | I21, I22 | Admission or outpatient department visit ≥1 with defined diagnosis code as primary-6^th^ diagnosis |
| Peripheral artery disease (PAD) | I70, I73 |  |
| Stroke | I63, I64 |  |
| Transient ischemic attack (TIA) | G458, G4599 |  |
| Thromboembolism | I74 |  |
| Dyslipidemia | E78 |  |
| Chronic lung disease (CLD) | J41-J44 |  |

The diagnosis was coded according to Korea Classification of Disease code (KCD code) modification of the International Classification of Disease-10 code (ICD-10 code). The 6th version of KCD code was applied to the data from August 1,2013 to December 31, 2015 and the 7th version of KCD code was applied to the data from January 1, 2016 to December 31, 2017 since the 6^th^ version of KCD code has been revised at January 01, 2016.

**Supplementary Table S3. Definitions of study outcomes**

| Outcome | Class | Code | Diagnostic definition |
| --- | --- | --- | --- |
| **Death** |  |  |  |
| All-cause death | Diagnosis | I46.1, R96.x, R98.x, R99.x | Claim ≥1 with relevant diagnosis code as primary or secondary diagnosis, or ‘dead’ claims in treatment result code |
|  | Treatment result code | 4 |  |
| CV-death |  |  |  |
| Rheumatic heart diseases | Diagnosis | I01.x, I02.0, I05.x-I09.x | Death claim with defined diagnosis code as primary diagnosis |
| Hypertensive diseases | Diagnosis | I10.x -I15.x |  |
| Ischemic heart diseases | Diagnosis | I20.x -I25.x |  |
| Myocardial infarction (MI) | Diagnosis | I21.x-I22.x |  |
| Pulmonary heart disease and diseases of pulmonary circulation | Diagnosis | I26.x, I27.x |  |
| Conduction disorders and arrhythmias | Diagnosis | I44.x, I45.x, I47.x, I48.x, I49.x |  |
| Heart failure | Diagnosis | I50.0, I50.1, I50.9 |  |
| Other heart diseases | Diagnosis | I30.x -I32.x, I33.x -I39.x, I40.x -I43.x, I51.x, I52.x |  |
| Ischemic stroke and transient ischemic attack (TIA) | Diagnosis | I63.x, I64.x, G45.x |  |
| Stroke | Diagnosis | I63.x, I64.x | Death claim with defined diagnosis code as primary diagnosis and procedure code |
|  | Procedure | HA441, HA451, HA461, HA851, HE101, HE201, HE301, HE401, HE501 |  |
| Atherosclerosis and arterial thromboembolism | Diagnosis | I67.2, I70.x, I74.x | Death claim with defined diagnosis code as primary diagnosis |
| Nonfatal cardiac arrest |  | I46.0 |  |
| Non-CV death |  |  | Death claim with any other primary diagnosis listed as CV-death |
| **Hospitalization** |  |  |  |
| CV-hospitalization |  |  |  |
| Rheumatic heart diseases | Diagnosis | I01.x, I02.0, I05.x-I09.x | Admission ≥1 (staying ≥1 day) with defined diagnosis code as primary diagnosis |
| Hypertensive diseases | Diagnosis | I10.x -I15.x |  |
| Ischemic heart diseases | Diagnosis | I20.x -I25.x |  |
| Myocardial infarction (MI) | Diagnosis | I21.x-I22.x |  |
| Pulmonary heart disease and diseases of pulmonary circulation | Diagnosis | I26.x, I27.x |  |
| Conduction disorders and arrhythmias | Diagnosis | I44.x, I45.x, I47.x, I48.x, I49.x |  |
| Heart failure | Diagnosis | I50.0, I50.1, I50.9 |  |
| Other heart diseases | Diagnosis | I30.x -I32.x, I33.x -I39.x, I40.x -I43.x, I51.x, I52.x |  |
| Ischemic stroke and transient ischemic attack (TIA) | Diagnosis | I63.x, I64.x, G45.x |  |
| Stroke | Diagnosis | I63.x, I64.x | Admission ≥1 (staying ≥3 days) and brain imaging (CT or MRI) with defined diagnosis code as primary diagnosis |
|  | Procedure | HA441, HA451, HA461, HA851, HE101, HE201, HE301, HE401, HE501 |  |
| Atherosclerosis and arterial thromboembolism | Diagnosis | I67.2, I70.x, I74.x | Admission ≥1 (staying ≥1 day) with defined diagnosis code as primary diagnosis |
| Nonfatal cardiac arrest | Diagnosis | I46.0 |  |
| Non-CV hospitalization |  |  | Admission ≥1 (staying ≥1 day) with any other primary diagnosis listed as CV-hospitalization |
| Hospitalization for potential safety signal |  |  |  |
| Hepatic disorder | Diagnosis | B15.x, B16.x, B17.x, B18.x, B19.x, K70.x, K71.x, K72.x, K73.x, K74.x, K75.x, K76.x, K77.x | Admission ≥1 (staying ≥1 day) with defined diagnosis code as primary diagnosis |
| Thyroid disorder | Diagnosis | E03.x, E05.x, J70.2 |  |
| Interstitial lung disease (ILD) | Diagnosis | J70.3, J70.x, J84.x |  |
| **Myocardial infarction (MI)** |  |  |  |
| MI | Diagnosis | I21.x-I22.x | Admission ≥1 (staying ≥1 day) with defined diagnosis code as primary diagnosis |
| **Stroke** |  |  |  |
| Stroke | Diagnosis | I63.x, I64.x | Admission ≥1 (staying ≥3 days) and brain imaging (CT or MRI) with defined diagnosis code as primary diagnosis |
|  | Procedure | HA441, HA451, HA461, HA851, HE101, HE201, HE301, HE401, HE501 |  |
| **Rhythm control** |  |  |  |
| Catheter Ablation | Procedure | M6544, M6545, M6549, M6540, M6541, M6542, M6546, M6547 | Claim ≥1 with defined procedure code |
| Electrical Cardioversion | Procedure | M5880 | Claim ≥1 with defined procedure code |
| Acute chemical cardioversion | Drug | Amiodarone (107402BIJ, 107430BIJ), Flecainide (501901BIJ, 159330BIJ, 501902BIJ, 159331BIJ) | Claim ≥1 with defined drug code |
| Non-pharmacological rhythm control |  | Catheter ablation + Electrical cardioversion | Each definition was described as above |
| Advanced rhythm control |  | Catheter ablation + Electrical cardioversion + Acute chemical cardioversion | Each definition was described as above |

The diagnosis was coded according to Korea Classification of Disease code (KCD code) modification of the International Classification of Disease-10 code (ICD-10 code). The 6th version of KCD code was applied to the data from August 1,2013 to December 31, 2015 and the 7th version of KCD code was applied to the data from January 1, 2016 to December 31, 2017 since the 6^th^ version of KCD code has been revised at January 01, 2016. The drug was coded according to Health Insurance Review and Assessment’s molecule code. The procedure was coded according to Health Insurance Review and Assessment reimbursed code.

**Supplementary Table S4. Crude and weighted incidence rates (per 100-person years) of clinical outcomes**

|  | **Crude incidence rate** | | | | | |  | **IPTW weighted incidence rate** | | | | | |
| --- | --- | --- | --- | --- | --- | --- | --- | --- | --- | --- | --- | --- | --- |
|  | **Dronedarone (n=3,119)** | |  | **Sotalol**  **(n=1,575)** | | **P-value** |  | **Dronedarone (n=3,123)** | |  | **Sotalol**  **(n=1,571)** | | **P-value** |
| **Outcomes** | **No. of patient with event (n)** | **Incidence rate per 100 PYs** |  | **No. of patient with event (n)** | **Incidence rate per 100 PYs** |  |  | **No. of patient with event (n)** | **Incidence rate per 100 PYs** |  | **No. of patient with event (n)** | **Incidence rate per 100 PYs** |  |
| **Primary outcome** |  |  |  |  |  |  |  |  |  |  |  |  |  |
| **Composite of CV hospitalization and all-cause death** | **466** | **16.72** |  | **307** | **30.71** | **<0.001** |  | **468** | **17.07** |  | **305** | **30.66** | **<0.001** |
| **Secondary outcomes** |  |  |  |  |  |  |  |  |  |  |  |  |  |
| **All-cause hospitalization** | **932** | **39.83** |  | **464** | **53.29** | **<0.001** |  | **915** | **39.68** |  | **473** | **55.06** | **<0.001** |
| **CV hospitalization** | **453** | **16.24** |  | **303** | **30.30** | **<0.001** |  | **455** | **16.62** |  | **301** | **30.24** | **<0.001** |
| Rheumatic heart diseases | 4 | 0.13 |  | 2 | 0.17 | 0.738 |  | 4 | 0.14 |  | 1 | 0.11 | 0.854 |
| Hypertensive diseases | 12 | 0.39 |  | 7 | 0.61 | 0.349 |  | 11 | 0.36 |  | 11 | 1.00 | 0.015 |
| Ischemic heart diseases | 98 | 3.28 |  | 34 | 3.03 | 0.686 |  | 93 | 3.15 |  | 35 | 3.10 | 0.939 |
| Myocardial infarction | 9 | 0.29 |  | 2 | 0.17 | 0.504 |  | 8 | 0.25 |  | 2 | 0.20 | 0.743 |
| Pulmonary heart diseases | 4 | 0.13 |  | 2 | 0.17 | 0.737 |  | 4 | 0.15 |  | 3 | 0.23 | 0.559 |
| Conduction disorders and arrhythmias | 276 | 9.44 |  | 213 | 20.05 | <0.001 |  | 283 | 9.84 |  | 202 | 19.01 | <0.001 |
| Heart failure | 16 | 0.52 |  | 9 | 0.78 | 0.330 |  | 16 | 0.53 |  | 14 | 1.21 | 0.024 |
| Other heart diseases | 28 | 0.91 |  | 21 | 1.85 | 0.015 |  | 30 | 1.01 |  | 21 | 1.89 | 0.027 |
| Ischemic stroke and transient ischemic attack (TIA) | 49 | 1.61 |  | 35 | 3.10 | 0.003 |  | 50 | 1.67 |  | 35 | 3.12 | 0.004 |
| Stroke | 26 | 0.85 |  | 17 | 1.49 | 0.072 |  | 26 | 0.85 |  | 18 | 1.57 | 0.044 |
| Atherosclerosis and arterial thromboembolism (TE) | 3 | 0.10 |  | 1 | 0.09 | 0.920 |  | 4 | 0.12 |  | 1 | 0.08 | 0.727 |
| Nonfatal cardiac arrest | 1 | 0.03 |  | 1 | 0.09 | 0.487 |  | 1 | 0.03 |  | 1 | 0.10 | 0.381 |
| **Non-CV hospitalization** | **628** | **25.13** |  | **233** | **23.73** | **0.454** |  | **612** | **24.84** |  | **241** | **24.65** | **0.918** |
| Senile cataract | 76 | 2.54 |  | 27 | 2.39 | 0.790 |  | 76 | 2.57 |  | 28 | 2.47 | 0.860 |
| Pneumonia, organism unspecified | 25 | 0.82 |  | 10 | 0.87 | 0.858 |  | 26 | 0.85 |  | 10 | 0.86 | 0.984 |
| Infectious gastroenteritis and colitis unspecified | 23 | 0.75 |  | 7 | 0.61 | 0.626 |  | 21 | 0.69 |  | 6 | 0.50 | 0.493 |
| Pain in throat and chest | 24 | 0.79 |  | 5 | 0.44 | 0.233 |  | 23 | 0.75 |  | 5 | 0.43 | 0.263 |
| Other spondylopathies | 17 | 0.56 |  | 9 | 0.78 | 0.405 |  | 16 | 0.55 |  | 11 | 0.92 | 0.185 |
| Hepatic disorder | 11 | 0.36 |  | 2 | 0.17 | 0.345 |  | 10 | 0.34 |  | 2 | 0.20 | 0.462 |
| Thyroid disorder | 1 | 0.03 |  | 0 | 0 | na |  | 1 | 0.03 |  | 0 | 0 | na |
| Interstitial lung diseases (ILD) | 2 | 0.07 |  | 0 | 0 | na |  | 2 | 0.05 |  | 0 | 0 | na |
| **All-cause death** | **21** | **0.68** |  | **8** | **0.70** | **0.965** |  | **20** | **0.66** |  | **10** | **0.83** | **0.564** |
| CV death | 2 | 0.07 |  | 3 | 0.26 | 0.128 |  | 2 | 0.07 |  | 4 | 0.34 | 0.059 |
| Non-CV death | 19 | 0.62 |  | 5 | 0.43 | 0.483 |  | 18 | 0.6 |  | 6 | 0.49 | 0.684 |
| **Stroke** | **26** | **0.85** |  | **17** | **1.49** | **0.072** |  | **26** | **0.85** |  | **18** | **1.57** | **0.044** |
| **Myocardial infarction** | **9** | **0.29** |  | **2** | **0.17** | **0.504** |  | **8** | **0.25** |  | **2** | **0.2** | **0.743** |
| **Exploratory outcomes** |  |  |  |  |  |  |  |  |  |  |  |  |  |
| **Non-pharmacological rhythm control** | **213** | **7.14** |  | **167** | **15.56** | **<0.001** |  | **224** | **7.66** |  | **154** | **14.25** | **<0.001** |
| Electrical cardioversion | 150 | 4.98 |  | 113 | 10.30 | <0.001 |  | 157 | 5.31 |  | 104 | 9.46 | <0.001 |
| Catheter ablation | 121 | 3.99 |  | 77 | 6.89 | <0.001 |  | 129 | 4.32 |  | 74 | 6.58 | 0.004 |
| **Advanced rhythm control** | **257** | **8.65** |  | **190** | **17.78** | **<0.001** |  | **267** | **9.16** |  | **179** | **16.66** | **<0.001** |
| Acute chemical cardioversion | 82 | 2.69 |  | 50 | 4.38 | 0.007 |  | 83 | 2.76 |  | 54 | 4.75 | 0.002 |
| Electrical cardioversion | 150 | 4.98 |  | 113 | 10.30 | <0.001 |  | 157 | 5.31 |  | 104 | 9.46 | <0.001 |
| Catheter ablation | 121 | 3.99 |  | 77 | 6.89 | <0.001 |  | 129 | 4.32 |  | 74 | 6.58 | 0.004 |

IPTW indicates inverse probability of treatment weight; CV, cardiovascular; MACE, major adverse cardiac event. IPTW was estimated using logistic regression with patient age, sex, CHA_2_DS_2_-VASc score, modified Charlson comorbidity index, number of baseline antiarrhythmic drugs, baseline antiarrhythmic drugs, comorbidities, concomitant drugs, hospital type at index date, insurance type and index year. P-value was derived from individual log-linked Poisson model

**Supplementary Table S5. Hazard ratio of hospitalization, death, and non-pharmacological rhythm control in dronedarone and sotalol groups**

|  | **Crude HR** | **P-value^b^** |  | **IPTW^a^ weighted HR** | **P-value^c^** |
| --- | --- | --- | --- | --- | --- |
| **Primary outcome** |  |  |  |  |  |
| **Composite of CV hospitalization and all-cause death** | **0.62 (0.53-0.71)** | **<0.001** |  | **0.63 (0.54-0.73)** | **<0.001** |
| **Secondary outcomes** |  |  |  |  |  |
| **All-cause hospitalization** | **0.82 (0.73 -0.91)** | **<0.001** |  | **0.79 (0.70 -0.88)** | **<0.001** |
| **CV hospitalization** | **0.61 (0.52 -0.70)** | **<0.001** |  | **0.62 (0.53 -0.72)** | **<0.001** |
| Rheumatic heart diseases | 0.80 (0.14 -4.43) | 0.796 |  | 1.25 (0.17 -9.05) | 0.826 |
| Hypertensive diseases | 0.70 (0.27 -1.80) | 0.460 |  | 0.40 (0.17 -0.92) | 0.032 |
| Ischemic heart diseases | 1.20 (0.81 -1.78) | 0.363 |  | 1.11 (0.75 -1.65) | 0.596 |
| Myocardial infarction | 1.81 (0.39-8.39) | 0.450 |  | 1.39 (0.31-6.13) | 0.668 |
| Pulmonary heart diseases | 0.86 (0.16 -4.70) | 0.860 |  | 0.70 (0.15 -3.21) | 0.645 |
| Conduction disorders and arrhythmias | 0.54 (0.45 -0.64) | <0.001 |  | 0.59 (0.49 -0.70) | <0.001 |
| Heart failure | 0.69 (0.30 -1.57) | 0.373 |  | 0.46 (0.22 -0.95) | 0.036 |
| Other heart diseases | 0.56 (0.31 -0.98) | 0.043 |  | 0.61 (0.35 -1.06) | 0.081 |
| Ischemic stroke and transient ischemic attack | 0.56 (0.36-0.86) | 0.009 |  | 0.58 (0.37-0.89) | 0.013 |
| Stroke | 0.59 (0.32-1.10) | 0.098 |  | 0.56 (0.30-1.03) | 0.062 |
| Atherosclerosis and arterial thromboembolism | 0.93 (0.09 -9.30) | 0.947 |  | 1.22 (0.12 -12.54) | 0.868 |
| Nonfatal cardiac arrest | 0.47 (0.03 -7.53) | 0.594 |  | 0.37 (0.02 -5.70) | 0.474 |
| **Non-CV hospitalization** | **1.11 (0.96 -1.29)** | **0.172** |  | **1.05 (0.91 -1.22)** | **0.491** |
| Senile cataract | 1.05 (0.67 -1.63) | 0.839 |  | 1.02 (0.66 -1.59) | 0.915 |
| Pneumonia, organism unspecified | 1.06 (0.51 -2.20) | 0.887 |  | 1.13 (0.54 -2.36) | 0.750 |
| Infectious gastroenteritis and colitis unspecified | 1.28 (0.55 -3.01) | 0.564 |  | 1.43 (0.56 -3.61) | 0.453 |
| Pain in throat and chest | 1.80 (0.68 -4.74) | 0.236 |  | 1.74 (0.65 -4.64) | 0.268 |
| Other spondylopathies | 0.67 (0.29 -1.50) | 0.327 |  | 0.56 (0.26 -1.22) | 0.146 |
| Hepatic disorder | 2.20 (0.48 -9.95) | 0.308 |  | 1.71 (0.41 -7.19) | 0.462 |
| Thyroid disorder | na |  |  | na |  |
| Interstitial lung diseases | na |  |  | na |  |
| **All-cause death** | **1.11 (0.49 -2.52)** | **0.800** |  | **0.88 (0.41 -1.91)** | **0.752** |
| CV death | 0.29 (0.05 -1.76) | 0.180 |  | 0.23 (0.04 -1.25) | 0.089 |
| Non-CV death | 1.60 (0.60 -4.29) | 0.352 |  | 1.33 (0.52 -3.44) | 0.555 |
| **Stroke** | **0.59 (0.32-1.10)** | **0.098** |  | **0.56 (0.30-1.03)** | **0.062** |
| **Myocardial infarction** | **1.81 (0.39-8.39)** | **0.450** |  | **1.39 (0.31-6.13)** | **0.668** |
| **Exploratory outcomes** |  |  |  |  |  |
| **Non-pharmacological rhythm control** | **0.54 (0.44 -0.66)** | **<0.001** |  | **0.63 (0.51 -0.77)** | **<0.001** |
| Electrical cardioversion | 0.57 (0.45 -0.73) | <0.001 |  | 0.66 (0.51 -0.84) | 0.001 |
| Catheter ablation | 0.64 (0.48 -0.85) | 0.002 |  | 0.72 (0.54 -0.96) | 0.026 |
| **Advanced rhythm control** | **0.56 (0.47-0.68)** | **<0.001** |  | **0.63 (0.52-0.76)** | **<0.001** |
| Acute chemical cardioversion | 0.69 (0.48-0.98) | 0.039 |  | 0.65 (0.46-0.92) | 0.014 |
| Electrical cardioversion | 0.57 (0.45 -0.73) | <0.001 |  | 0.66 (0.51 -0.84) | 0.001 |
| Catheter ablation | 0.64 (0.48 -0.85) | 0.002 |  | 0.72 (0.54 -0.96) | 0.026 |

IPTW indicates inverse probability of treatment weight; HR, hazard ratio; CI, confidence interval; CV, cardiovascular; na, not applicable; compared with sotalol group as the reference.

a: IPTW was estimated using logistic regression with patient age, sex, CHA_2_DS_2_-VASc score, mCCI, number of AADs in baseline, AADs in baseline, comorbidity, concomitant drugs, hospital type at index date, insurance type and index year. b: P-value was derived from individual cox-proportional hazard regression. c: P-value was derived from individual cox-proportional hazard regression with IPTW

**Supplementary Tabe S6. Each component of conduction disorders and arrhythmias**

|  | **Crude event number (IR)** | | **Crude HR** | **p-value** | **IPTW event number (IR)** | | **IPTW HR** | **p-value** |
| --- | --- | --- | --- | --- | --- | --- | --- | --- |
|  | **Dronedarone**  **(n=3,119)** | **Sotalol**  **(n=1,575)** |  |  | **Dronedarone**  **(n=3,123)** | **Sotalol**  **(n=1,571)** |  |  |
| **Conduction disorders and arrhythmias** | 276 (9.44) | 213 (20.05) | 0.54 (0.45-0.64) | <0.001 | 283 (9.84) | 202 (19.01) | 0.59 (0.49-0.70) | <0.001 |
| **Classification by ICD codes** |  |  |  |  |  |  |  |  |
| I44.x (AV or LBB block) | 2 (0.07) | 4 (0.35) | 0.22 (0.04-1.23) | 0.0847 | 2 (0.06) | 4 (0.36) | 0.21 (0.04-1.18) | 0.0762 |
| I45.x (other conduction disorders) | 1 (0.03) | 1 (0.09) | 0.49 (0.03-7.88) | 0.6169 | 1 (0.05) | 1 (0.06) | 1.01 (0.06-16.9) | 0.9972 |
| I47.x (paroxysmal tachycardia) | 5 (0.16) | 9 (0.78) | 0.22 (0.07-0.68) | 0.0080 | 5 (0.18) | 10 (0.90) | 0.22 (0.08-0.62) | 0.0043 |
| I48.x (atrial fibrillation and flutter) | 256 (8.71) | 191 (17.81) | 0.55 (0.46-0.67) | <0.0001 | 263 (9.12) | 180 (16.69) | 0.62 (0.51-0.74) | <0.0001 |
| I49.x (other cardiac arrhythmias) | 17 (0.56) | 12 (1.05) | 0.64 (0.30-1.34) | 0.2319 | 15 (0.51) | 12 (1.03) | 0.59 (0.28-1.27) | 0.1779 |
| **Clinical classification** |  |  |  |  |  |  |  |  |
| Atrial fibrillation (I48.x) | 256 (8.71) | 191 (17.81) | 0.55 (0.46-0.67) | <0.0001 | 263 (9.12) | 180 (16.69) | 0.62 (0.51-0.74) | <0.0001 |
| Bradycardia/SSS (R00.1+I49.5) | 10 (0.33) | 7 (0.61) | 0.63 (0.24-1.67) | 0.3561 | 9 (0.29) | 8 (0.70) | 0.49 (0.19-1.28) | 0.1462 |
| AV block (I44.2+I44.3) | 1 (0.03) | 4 (0.35) | 0.12 (0.01-1.05) | 0.0550 | 1 (0.03) | 4 (0.36) | 0.12 (0.01-1.05) | 0.0550 |
| Ventricular tachyarrhythmia  (I47.2+I49.0) | 1 (0.03) | 7 (0.61) | 0.06 (0.01-0.48) | 0.0080 | 1 (0.03) | 8 (0.73) | 0.04 (0.00-0.40) | 0.0062 |

Abbreviations: AV, atrioventricular; HR, hazard ratio; IPTW, inverse probability of treatment weighting; IR, incidence rate; LBB, left bundle branch; SSS, sick sinus syndrome.

**Supplementary Table S7. Persistence and discontinuation pattern including time on treatment**

|  | **All**  **(N=4,694)** |  | **Dronedarone**  **(N=3,119)** |  | **Sotalol**  **(N=1,575)** |  | **P-value** |
| --- | --- | --- | --- | --- | --- | --- | --- |
| **Persistence rate & discontinuation pattern (n, %)** |  |  |  |  |  |  |  |
| **Continuation** | 845 (18.0) |  | 656 (21.0) |  | 189 (12.0) |  | <0.001 |
| **Discontinuation** | 3,849 (82.0) |  | 2,463 (79.0) |  | 1,386 (88.0) |  | <0.001 |
| Stop | 1,073 (27.9) |  | 715 (29.0) |  | 358 (25.8) |  | 0.881 |
| Switch | 2,021 (52.5) |  | 1,232 (50.0) |  | 789 (56.9) |  | <0.001 |
| to [AMI] | 769 (38.1) |  | 457 (37.1) |  | 312 (39.5) |  | <0.001 |
| to [FLE] | 533 (26.4) |  | 333 (27.0) |  | 200 (25.3) |  | 0.039 |
| to [PRO] | 397 (19.6) |  | 272 (22.1) |  | 125 (15.8) |  | 0.362 |
| to [PIL] | 127 (6.3) |  | 96 (7.8) |  | 31 (3.9) |  | 0.027 |
| to [DRO] | 92 (4.6) |  | 9 (0.7) |  | 83 (10.5) |  | <0.001 |
| to [SOT] | 62 (3.1) |  | 47 (3.8) |  | 15 (1.9) |  | <0.001 |
| to [AMI+FLE] | 19 (0.9) |  | 8 (0.6) |  | 11 (1.4) |  | 0.024 |
| to [AMI+PRO] | 12 (0.6) |  | 7 (0.6) |  | 5 (0.6) |  | 0.551 |
| to [FLE+SOT] | 3 (0.1) |  | 1 (0.1) |  | 2 (0.3) |  | <0.001 |
| to [PRO+SOT] | 2 (0.1) |  | 0 (0.0) |  | 2 (0.3) |  | na |
| to [AMI+FLE+PRO] | 1 (0.0) |  | 0 (0.0) |  | 1 (0.1) |  | na |
| to [AMI+PIL] | 1 (0.0) |  | 1 (0.1) |  | 0 (0.0) |  | na |
| to [FLE+PRO] | 1 (0.0) |  | 1 (0.1) |  | 0 (0.0) |  | na |
| to [PIL+PRO] | 1 (0.0) |  | 0 (0.0) |  | 1 (0.1) |  | na |
| to [PIL+SOT] | 1 (0.0) |  | 0 (0.0) |  | 1 (0.1) |  | na |
| Restart | 654 (17.0) |  | 446 (18.1) |  | 208 (15.0) |  | 0.307 |
| Add-on | 101 (2.2) |  | 70 (2.2) |  | 31 (2.0) |  | 0.538 |
| **Time on treatment (days, mean±SD)** |  |  |  |  |  |  |  |
| All patients | 335.2±372.9 |  | 368.0±397.9 |  | 270.2±307.4 |  | <0.001 |
| Continued patients | 863.1±362.3 |  | 888.0±366.1 |  | 776.7±336.0 |  | <0.001 |
| Discontinued patients | 219.3±257.2 |  | 229.5±271.4 |  | 201.1±228.7 |  | <0.001 |
| Stop | 206.7±241.7 |  | 202.8±244.8 |  | 214.4±235.5 |  | 0.451 |
| Switch | 189.7±235.1 |  | 197.4±250.7 |  | 177.8±207.9 |  | 0.058 |
| Restart | 330.9±315.3 |  | 359.2±327.5 |  | 270.2±278.6 |  | <0.001 |
| Add-on | 221.5±227.9 |  | 242.0±243.7 |  | 175.3±182.6 |  | 0.133 |

AMI indicates amiodarone; FLE, flecainide; PRO, propafenone; PIL, pilsicainide; na, not applicable. P-value was derived from individual chi-square test for number of patients by persistence group and t-test for time on treatment days

**Supplementary Table S8. Adherence rate and medication possession rate (MPR)**

|  | **All**  **(N=4,694)** |  | **Dronedarone**  **(N=3,119)** |  | **Sotalol**  **(N=1,575)** |  | **P-value** |
| --- | --- | --- | --- | --- | --- | --- | --- |
| **Adherence rate (n, %)** |  |  |  |  |  |  |  |
| MPR ≥ 0.8 | 4,652 (99.1) |  | 3,087 (99.0) |  | 1,565 (99.4) |  | 0.179 |
| MPR < 0.8 | 42 (0.9) |  | 32 (1.0) |  | 10 (0.6) |  |  |
| **MPR (mean±SD)** |  |  |  |  |  |  |  |
| All patients | 1.03±0.10 |  | 1.02±0.09 |  | 1.03±0.11 |  | 0.006 |
| Continued patients | 1.10±0.13 |  | 1.09±0.12 |  | 1.13±0.16 |  | 0.002 |
| Discontinued patients | 1.01±0.09 |  | 1.01±0.08 |  | 1.02±0.10 |  | <0.001 |
| Stop | 1.01±0.09 |  | 1.00±0.09 |  | 1.02±0.10 |  | 0.082 |
| Switch | 1.02±0.08 |  | 1.01±0.07 |  | 1.02±0.09 |  | 0.001 |
| Restart | 1.01±0.09 |  | 1.00±0.09 |  | 1.02±0.11 |  | 0.028 |
| Add-on | 1.02±0.09 |  | 1.02±0.08 |  | 1.02±0.12 |  | 0.946 |

MPR indicates medication possession ratio. P-value was derived from individual chi-square test for number of patients by persistence group and t-test for MPR

**IV. Supplementary Figures**

**Supplementary Figure S1. Study enrollment flow**

Patients who were prescribed with dronedarone or sotalol with ≥ 7 days of supply from 01 August 2013

to 31 December 2019 (n=10,229)

Patients who were diagnosed with atrial fibrillation during 36-months prior to the index date

(n=8,574)

Dronedarone group

(n=3,119)

Sotalol group

(n=1,575)

Excluded those who were:

- Aged < 18 years as of the index date (n=20)
- Diagnosed with cancer and end renal disease(ESRD) during the study period (n=922)
- Exposed to dronedarone or sotalol for ≥7 days during the 12-month baseline period prior to the index date (n=1,206)
- Diagnosed with contraindication of dronedarone or sotalol during the baseline period, such as heart failure, sick sinus syndrome or II- or III-degree atrioventricular block, bradycardia, long QT syndrome, severe hepatic impairment, pregnancy, cardiogenic shock, hypokalemia, bronchial asthma (n=1,328)
- Prescribed with contraindicated medication of dronedarone or sotalol during the baseline or follow-up period, such as strong CYP3A inhibitors, phenothiazine, tricyclic antidepressants, macrolide antibiotics, combination of class Ic and III antiarrhythmics combination (n=404)

**Supplementary Figure S2. PS distribution of dronedarone and sotalol groups before and after IPTW**

| 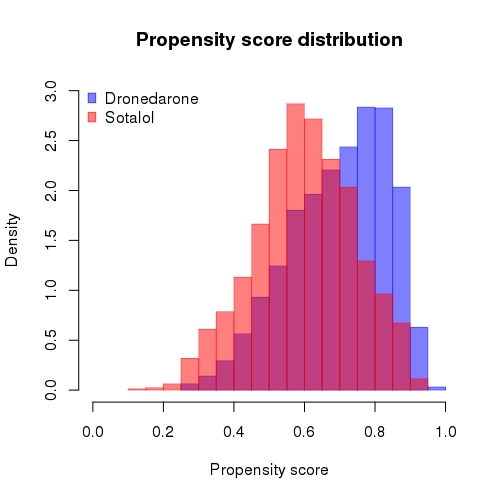 | 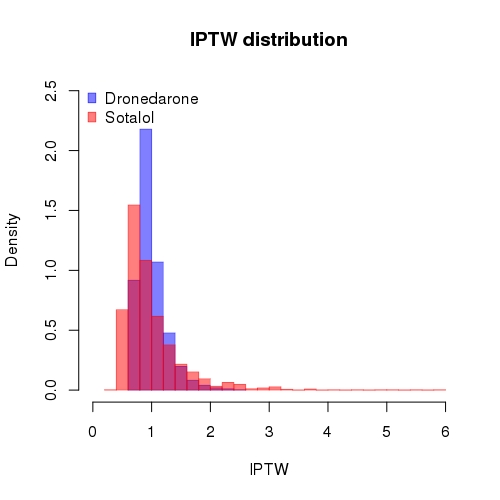 |
| --- | --- |

**Supplementary Figure S3. Weighted cumulative incidence curves of hospitalization, death, stroke, and myocardial infarction in dronedarone and sotalol groups**

| A. All-cause hospitalization | B. CV hospitalization | C. Non-CV hospitalization |
| --- | --- | --- |
| 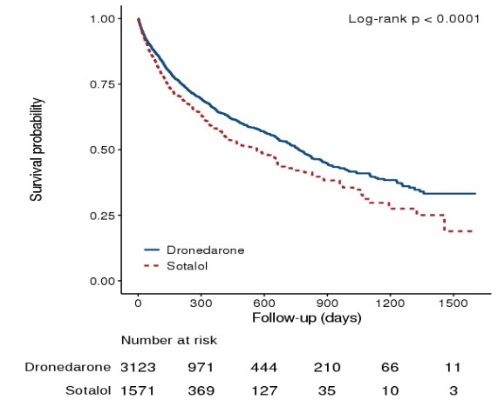 | 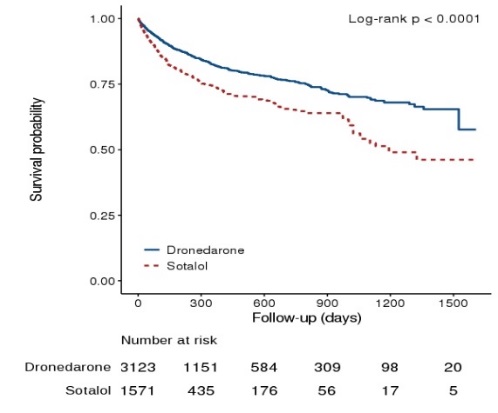 | 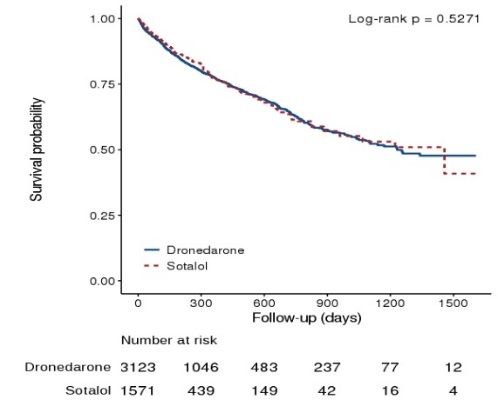 |
| D. All-cause death | E. CV death | F. Non-CV death |
| 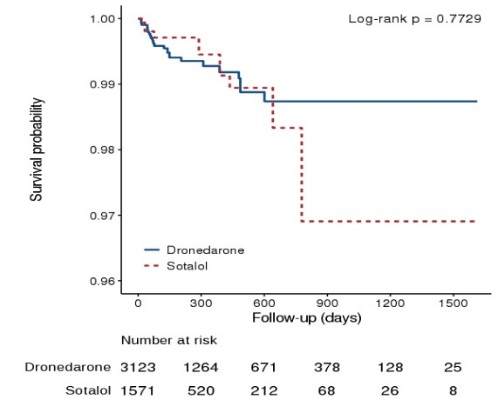 | 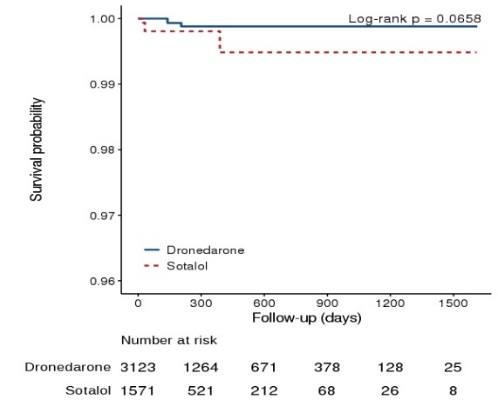 | 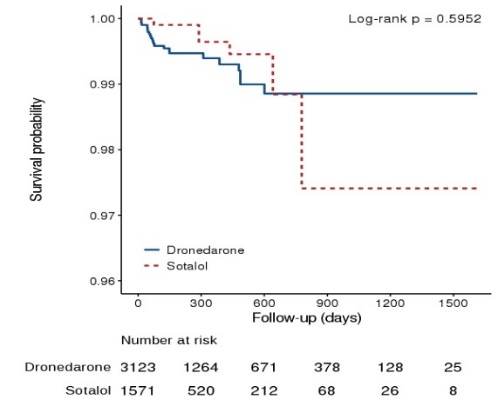 |
| G. Stroke | H. Myocardial infarction |  |
| 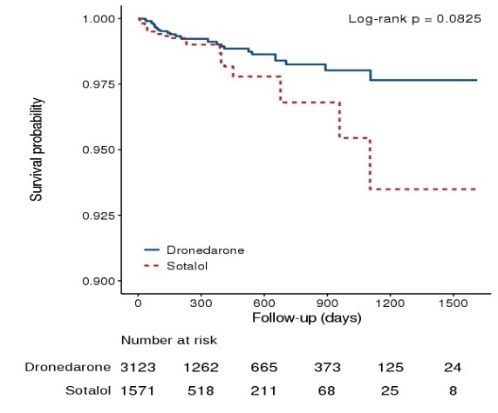 | 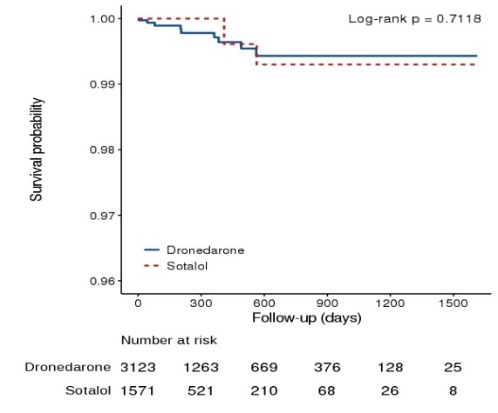 |  |

**Supplementary Figure S4. Weighted cumulative incidence curves of hospitalization due to conduction disorders and arrhythmia, ischemic stroke and transient ischemic attack, and heart failure in dronedarone and sotalol groups**

| A. Conduction disorders and arrhythmia | B. Ischemic stroke and TIA | C. Heart failure |
| --- | --- | --- |
| 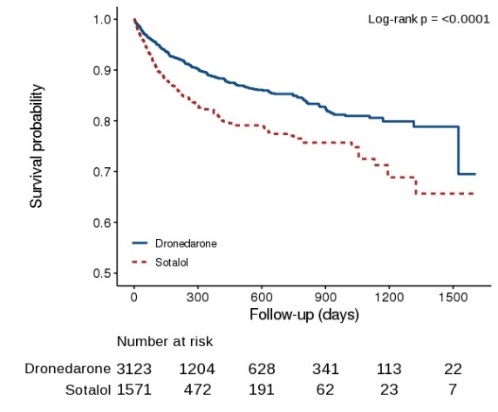 | 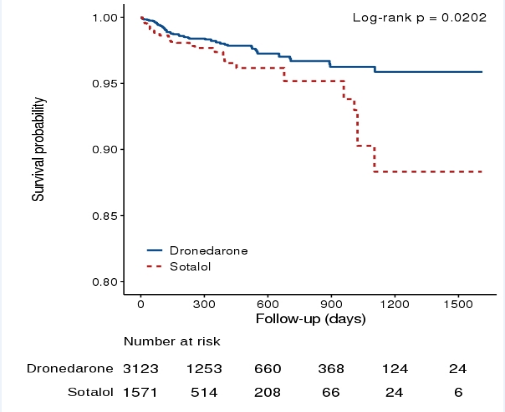 | 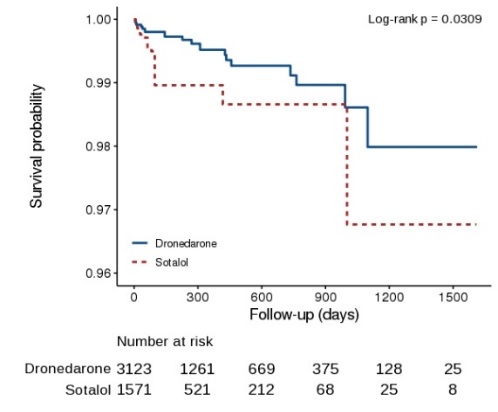 |

Abbreviation: TIA, transient ischemic attack
